# Shigella-Controlled Human Infection Models: Current and Future Perspectives

**DOI:** 10.1007/82_2021_248

**Published:** 2024-01-01

**Authors:** Kristen A. Clarkson, Chad K. Porter, Kawsar R. Talaat, Melissa C. Kapulu, Wilbur H. Chen, Robert W. Frenck, A. Louis Bourgeois, Robert W. Kaminski, Laura B. Martin

**Affiliations:** Department of Diarrheal Disease Research, Walter Reed Army Institute of Research, 503 Robert Grant Avenue, Silver Spring, MD 20910, USA; Enteric Disease Department, Naval Medical Research Center, 503 Robert Grant Avenue, Silver Spring, MD 20910, USA; Center for Immunization Research, Johns Hopkins Bloomberg School of Public Health, 624 North Broadway Street Hampton House, Baltimore, MD 21205, USA; Center for Immunization Research, Johns Hopkins Bloomberg School of Public Health, 624 North Broadway Street Hampton House, Baltimore, MD 21205, USA; Center for Vaccine Development and Global Health, University of Maryland School of Medicine, 685 West Baltimore Street, Baltimore, MD 21201, USA; Cincinnati Children’s Hospital Medical Center, 3333 Burnet Avenue, Cincinnati, OH 45229, USA; PATH Center for Vaccine Innovation and Access, 455 Massachusetts Avenue NW, Washington, DC 20001, USA; Department of Diarrheal Disease Research, Walter Reed Army Institute of Research, 503 Robert Grant Avenue, Silver Spring, MD 20910, USA; GSK Vaccines Institute for Global Health, Via Fiorentina 1, 53100 Siena, Italy

## Abstract

*Shigella*-controlled human infection models (CHIMs) are an invaluable tool utilized by the vaccine community to combat one of the leading global causes of infectious diarrhea, which affects infants, children and adults regardless of socioeconomic status. The impact of shigellosis disproportionately affects children in low- and middle-income countries (LMICs) resulting in cognitive and physical stunting, perpetuating a cycle that must be halted. *Shigella*-CHIMs not only facilitate the early evaluation of enteric countermeasures and up-selection of the most promising products but also provide insight into mechanisms of infection and immunity that are not possible utilizing animal models or in vitro systems. The greater understanding of shigellosis obtained in CHIMs builds and empowers the development of new generation solutions to global health issues which are unattainable in the conventional laboratory and clinical settings. Therefore, refining, mining and expansion of safe and reproducible infection models hold the potential to create effective means to end diarrheal disease and associated co-morbidities associated with *Shigella* infection.

## Abbreviations

AMR: Antimicrobial resistance
AR: Attack rate
CCHMC: Cincinnati Children’s Hospital Medical Center
CDC: US Center for Disease Control and prevention
CFU: Colony forming units
CHIM: Controlled human infection model
CoP: Correlates of protection
CRP: C-reactive protein
EE: Environmental enteropathy
EMA: European Medicines Agency
ETEC: Enterotoxigenic *Escherichia coli*
FDA: US Food and Drug Administration
G4C: Group 4 capsule
GMP: Good manufacturing practices
HBD-1: Human β-defensin-1
HICs: High-income countries
IABS: International Alliance for Biological Standardisation
Ipa: Invasion plasmid antigens
LMICs: Low- and middle-income countries
LPS: Lipopolysaccharide
mAb: Monoclonal antibody
MPO: Myeloperoxidase
OAg: O-antigen
SAGE: Strategic Advisory Group of Experts
T3SS: Type-III secretion system
T6SS: Type-VI secretion system
TCV: Typhoid conjugate vaccine
US: United States
WHO: World Health Organization

## 1 Introduction

### 1.1 Shigella Disease Burden

Based on current estimates, 500,000 children under 5 years old die annually worldwide from diarrheal diseases, while millions more suffer multiple episodes during early childhood (GBD 2016 Diarrhoeal Disease Collaborators 2018). Our understanding of the global burden of Shigella-related disease has been greatly enhanced in recent years by the results of several large-scale epidemiological studies in South Asia, Africa and South America and by the application of new molecular-based diagnostics for case detection in both case-control and community-based prospective surveillance studies (GBD 2016 Diarrhoeal Disease Collaborators 2018; Livio et al. 2014; Liu et al. 2016; Tickell et al. 2017; WHO 2020). *Shigella* is the most common bacterial cause of moderate to severe diarrhea (bloody and non-bloody) in children under 5 years of age, with the current trends indicating the highest incidence occurring among children in their second year of life living in Africa and South Asia (Ahmed et al. 1997; Rogawski McQuade et al. 2020). The high burden of *Shigella* infections early in life has also been associated with the subsequent development of environmental enteropathy (EE) which can manifest as a “triple burden” of sequelae: (1) increased stunting-associated risk of mortality due to other infectious diseases, (2) poor cognitive development and subsequent reduction in educational outcomes and contribution to human capital and (3) increased risk of non-communicable diseases as adults (Guerrant et al. 2013, 2021; Lamberti et al. 2014; Rogawski et al. 2018; Anderson et al. 2019a, b). There is also the potential for post-infectious sequelae such as reactive arthritis and irritable bowel syndrome (Pogreba-Brown et al. 2020).

In addition to its impact in endemic settings, *Shigella*-attributable diarrhea among travelers and military populations can cause significant morbidity and incapacitation, requiring antibiotics, intravenous fluids and hospitalization (Riddle et al. 2006; Shah et al. 2009; Steffen 2017; Riddle 2018; Olson et al. 2019). *Shigella* is also considered an antimicrobial resistance (AMR) threat by the World Health Organization (WHO) and the US Center for Disease Control (CDC) (WHO 2017a; CDC 2019), leading the WHO Global Antimicrobial Resistance Surveillance System to identify *Shigella* as a priority pathogen for the development of new interventions. The WHO, in conjunction with the Wellcome Trust and Boston Consulting Group (Wellcome Trust and Boston Consulting Group 2021), has recommended acceleration of vaccine development for *Shigella* due to its high global burden of illness and the associated difficulty in effective treatment with commonly available antibiotics (CDC 2019).

The need to accelerate *Shigella* vaccine development has caused stakeholders, donors and the *Shigella* vaccine development community to consider how CHIMs could contribute to accelerated licensure of *Shigella* vaccines. CHIMs have been established for a broad range of enteric pathogens, including *Shigella,* and can provide insight into disease pathogenesis or the immune response to infection. Albeit in a small number of individuals, CHIMs can also be used to assess the early clinical efficacy of candidate vaccines and facilitate the identification of correlates of protection (CoP) by evaluating immune responses associated with protective efficacy (Holmgren et al. 2017; Giersing et al. 2019; MacLennan et al. 2019a; Clarkson et al. 2021a). For other enteric pathogens, CHIMs have been used to support licensure and recommendation by the WHO Strategic Advisory Group of Experts (SAGE) for vaccine uptake and expanded utilization (Giersing et al. 2019; MacLennan et al. 2019a).

The usefulness of *Shigella*-CHIMs is further solidified by the rapid expansion of Omics and systems biology technologies which can provide important information on host and bacterial factors that contribute to *Shigella*-associated disease and gut inflammation, as well as the cellular, molecular and biochemical basis of *Shigella*specific immunity. The challenges and potential benefits of the expanded use of standardized *Shigella*-CHIMs are further detailed below. This chapter also describes how host and bacterial factors can contribute to model consistency and how recent innovations align with existing animal models, as well as the newly developed in vitro human enteroid model. Finally, this chapter summarizes opportunities to expand challenge model capabilities to include new *Shigella* strains, clinical settings, microbiological and immunological outcomes and efforts to expand its application to LMICs and support policy recommendations for *Shigella* vaccine licensure, WHO prequalification and vaccine uptake in *Shigella* endemic areas.

### 1.2 Shigella *Species and Global Distribution*

The *Shigella* genus is currently classified into four species, each of which is human enteropathogens. These can be grouped based on serological reactivity with group-specific antisera into S. *dysenteriae* (serogroup A), S. *flexneri* (serogroup B), S. *boydii* (serogroup C) and S. *sonnei* (serogroup D). These bacteria can be further classified into serotypes, based on the chemical structures of the repeating saccharide units of the O-antigen (OAg), the surface exposed part of lipopolysaccharide (LPS) (Lindberg et al. 1991). However, the OAg of some *Shigella* species is structurally identical (Liu et al. 2008), serologically cross-react (Lefebvre et al. 1995) and shares gene clusters (Liu et al. 2008; Pupo et al. 2000; Yang et al. 2005) with some *Escherichia coli*, thus complicating the ability to distinguish these bacteria. Currently, there are at least 54 unique *Shigella* serotypes that are recognized (Muthuirulandi Sethuvel et al. 2017): 15 serotypes of S. *dysenteriae,* 19 serotypes and subserotypes of *S.* fl*exneri*, 19 serotypes of *S. boydii* and a single serotype of *S. sonnei*.

Among the *S.* fl*exneri* serogroup, there are molecular similarities between the structures of the LPS OAg repeating units. With the exception of *S.* fl*exneri* 6, there is a common linear tetrasaccharide repeating unit composed of three a-L-rhamnosyl residues and one residue of N-acetyl-D-glucosamine [*α*-L-Rha*p*-(1 → 2)-*α*-L-Rha*p*-(1 → 3)-*α*-L-Rha*p*-(1 → 3)-β-D-Gluc*p*NAc]; each of which is joined through a β-D-Gluc*p*NAc-(1 → 2)-*α*-L-Rha*p* linkage (Carlin et al. 1984; Kenne et al. 1978). Antigenic diversity among the *S.* fl*exneri* serogroup is due to additional *α*-D-glucopyranosyl and O-acetyl moieties at various positions on the tetrasaccharide subunit which are the basis for the designation of “type” (I, II, III, IV) and “group” (3,4; 6; 7,8) antigenic factors associated with S. *flexneri* serotypes (Perepelov et al. 2012; Knirel et al. 2015). Although *S. flexneri* 6 does not share the same linear tetrasaccharide repeating unit, the LPS does share a common *O*-acetylated disaccharide [*α*-L-Rha*p*-(1 → 2)-*α*-L-Rha*p*] with S. *flexneri* 2a.

Given that immunity to *Shigella* infection has been demonstrated to be serotype-specific, the generation of serotype-specific OAg-based immunity is the focus of many *Shigella* vaccines under development (Table 1). Nonetheless, other antigenic components may be important in *Shigella* pathogenesis. The virulence of *Shigella* is dependent upon the presence of a large virulence plasmid (>200 kbp) (Sansonetti et al. 1982), which encodes for the type-III secretion system (T3SS), and is necessary for the full pathogenicity of the bacterium (Schroeder and Hilbi 2008). Importantly, the T3SS syringe-like apparatus includes several invasion plasmid antigens (Ipa), which are shared across all invasive *Shigella* strains carrying the virulence plasmid. Other conserved virulence factors/antigens that are important in *Shigella* pathogenesis include VirG (IscA) (Shimanovich et al. 2017) and PSSP-1 (C-terminus of IcsP) (Kim et al. 2015). Both VirG and PSSP-1 proteins contribute to the cell-to-cell spread of *Shigella* among enterocytes in the large intestine and rectal mucosa.

The global distribution of *Shigella* species appears to follow a general trend that may reflect the predilection for certain species to have greater incidence according to the local availability of clean water, sanitation, hygiene and population nutritional status. Thus, S. *flexneri* represent the predominant species for the majority (>60%) of the burden of shigellosis in LMICs, whereas S. *sonnei* is the predominant species (>70%) afflicting high-income countries (HICs) (Livio et al. 2014; Gupta et al. 2004; Gu et al. 2012). Additionally, for countries that are economically transitioning from low- or middle-income to high-income status, these improvements have been associated with shifts away from *S. flexneri* toward *S. sonnei* predominance (Ram et al. 2008; Thompson et al. 2015; Qiu et al. 2015). An explanation for this observed epidemiological pattern is that individuals in LMICs have gained natural immunity to *S. sonnei* through frequent exposure to *Plesiomonas shigelloides,* which possesses a homologous LPS structure to S. *sonnei* and is often found in low-resource settings with poor infrastructure (Sack et al. 1994). Those living in higher income nations would not gain this natural immunity, as their improvements in infrastructure and sanitation measures have resulted in a low prevalence of *P*. *shigelloides*.

S. *dysenteriae* and S. *boydii* cause a minor proportion (≤ 5%) of overall shigellosis globally. In the past, large outbreaks of *S. dysenteriae* serotype A1 occurred generally once a decade in South Asia and central Africa particularly in refugee settings, where they were associated with a high case fatality rate and increasing antibiotic resistance. However, in recent years these outbreaks have inexplicably disappeared (Kerneis et al. 2009; Bardhan et al. 2010). The prevalent *Shigella* species, especially the *S. flexneri* serotypes, can be both temporally and geographically variable, as has been demonstrated over numerous attempts to characterize the epidemiology of shigellosis (Livio et al. 2014; Kotloff et al. 1999; Seidlein et al. 2006). Nonetheless, informed by epidemiological data on the prevalence of *Shigella* serogroups and serotypes, the largest proportion of the global disease burden has been attributed to the following serotypes: *S. sonnei* and *S. flexneri* 1b, 2a, 3a and 6 (Livio et al. 2014; Barry and Levine 2019). Multivalent vaccine approaches would be required to provide broad protection against these serogroups and serotypes, and others that may emerge (Seidlein et al. 2006).

### 1.3 S. sonnei *Is a Unique Serogroup Among* Shigella spp

Among the major *Shigella* serotypes associated with enteric illness, *S. sonnei* is unique for several reasons. *S. sonnei* encodes for a type-VI secretion system (T6SS) which is used to directly compete with and kill, not only other *Shigella* species but also other commensal enteric bacteria including *E. coli* (Anderson et al. 2017). Interestingly, the T6SS of *S. sonnei* has been cited as one of the reasons that *S. sonnei* has been able to rise to dominance in higher income countries. In the absence of natural immunity to *P*. *shigelloides*, *S. sonnei* has been able to out-compete other *Shigella* serotypes with the help of its T6SS (Sack et al. 1994). The T6SS can also play a role in altering the innate immune response and inflammatory environment during *Shigella* infection via the killing of intestinal microbiota and commensal microorganisms.

Other than *S. flexneri* 6, *S. sonnei* is also the only *Shigella* serotype known to express a group 4 capsule (G4C), characterized by structural similarity to the OAg (Caboni et al. 2015). The G4C is an important virulence factor, in that it creates a thick layer of polysaccharide on the surface of *S. sonnei* strains, reducing accessibility to the T3SS apparatus. The G4C also increases the resistance of *S. sonnei* to the bactericidal activity of antibodies (Caboni et al. 2015), which in conjunction with reduced cellular invasion creates a less inflammatory environment (Watson et al. 2019) and improves the ability of *S. sonnei* to persist in the human gut. Another unique attribute of *S. sonnei* is its adeptness in acquiring AMR genes compared to other *Shigella* serotypes. Thus, allowing *S. sonnei* to persist during infection and therapeutic treatment (Thompson et al. 2015).

### 1.4 *Current Status* of Shigella *Vaccine Development*

As shown in Table 1, the current portfolio of *Shigella* vaccine candidates target serotype-specific OAg alone (bioconjugates, synthetic conjugates and GMMA) or with conserved proteins (Invaplex) as well as multiple surface-expressed antigens (attenuated strains). These approaches target single serotypes, multiple serotypes and combinations of pathogens. At present, only single serotype vaccines have been evaluated in *Shigella*-CHIMs, revealing useful and unexpected data to further inform early clinical development. This expanded value of CHIMs has led to further efforts to harmonize the models and expand their application to LIMC settings. Similarly for ongoing and planned field evaluations of candidate vaccines in Kenya, sponsors and funders have harmonized the human trial protocols for clinical and immunologic readouts to streamline development among the most advanced OAg-based vaccines. In parallel, critical vaccine development efforts are ongoing to standardize the assays and tools as well as the optimal regulatory pathway and policy roadmap that will be needed for the roll-out and implementation of a safe and effective *Shigella* vaccine. The use of existing and new *Shigella*-CHIMs, both in HICs and their establishment in LMICs, will play a pivotal role.

## 2 Summary of Existing *Shigella-CHIMs*

The *Shigella* field currently utilizes two CHIMs developed for *S. flexneri* 2a and *S. sonnei*. To date, the majority of *Shigella* CHIMs have been conducted using the *S. flexneri* 2a model (Table 2). The limited experience with the *S. sonnei* CHIM has resulted in the use of outcome definitions, both for disease and immune responses, which were derived predominantly from *S. flexneri* 2a data. While many parameters of these two *Shigella*-CHIMs are similar, the evaluation of *S. flexneri* 2a and *S. sonnei* strains as challenge agents has shown differences in pathogenesis, translating into important distinctions in the spectrum of illness these strains induce (Clarkson et al. 2021b). Furthermore, evaluation of the immune responses induced by these two challenge agents has highlighted important differences in the magnitude of the innate inflammatory response, as well as the phenotype of the systemic and mucosal adaptive immune responses they trigger. The differences in immune response profiles may reflect different mechanisms of protection and immune CoP for shigellosis. By rigorously comparing and contrasting the key attributes of the *S. flexneri* 2a and *S. sonnei* CHIMs (outlined below), the field can be better informed of how new *Shigella*-CHIMs should be developed. It is likely that not all *Shigella* serotypes are created equal, and each new model will need to be treated as unique rather than as an extension of an existing model as was done for the *S. sonnei* (lyophilized) CHIM using S. *flexneri 2a* data. New *Shigella***-CHIMs** should be established de novo and well-characterized during clinical setup to mitigate against important unappreciated differences among *Shigella* spp.

### 2.1 Historical Context and Brief Overview of Established Models

The first experimental human *Shigella* challenge occurred in the 1940s using a *S. flexneri* strain that is no longer utilized (Shaughnessy and Olsson 1946). As late as the 1950s, despite multiple attempts, no effective vaccine against *Shigella* had been found, leading Colonel David Mel (Military Medical Academy, Yugoslavia) to conclude “protection against bacillary dysentery cannot be achieved by parenterally administered vaccines” (Mel et al. 1965a). Yet, his observations that “living cell” preparations administered by the oral route could achieve protection led to a series of field trials of live, attenuated, streptomycin-dependent *Shigella* vaccines (Mel et al. 1965b, c, 1968, 1971). These field trials in the 1960s were conducted in hyper-endemic regions in Yugoslavia and showed high efficacy in both adults (Mel et al. 1965c, 1968) and children (2–8 years of age) (Mel et al. 1971). An early live oral vaccine candidate requiring a 5-dose schedule (each dose spaced 3 days apart) elicited only short-lived immunity (Mel et al. 1974) and was found to be susceptible to genetic reversion (streptomycin-independence, but not invasion) (Levine et al. 1975). By the early 1970s, additional oral, live-attenuated *Shigella* vaccines were being evaluated (DuPont et al. 1972a, b; Levine et al. 1972, 1977). Using the established *Shigella*-CHIM (DuPont et al. 1969), positive efficacy was demonstrated in adults using a fully virulent *Shigella flexneri* 2a strain (DuPont et al. 1972b). These live-*Shigella* preparations used for oral vaccination could be considered early *Shigella*-CHIMs and could help to support the use of safer attenuated *Shigella* strains in experimental humans, if required.

**Fig. 1:**
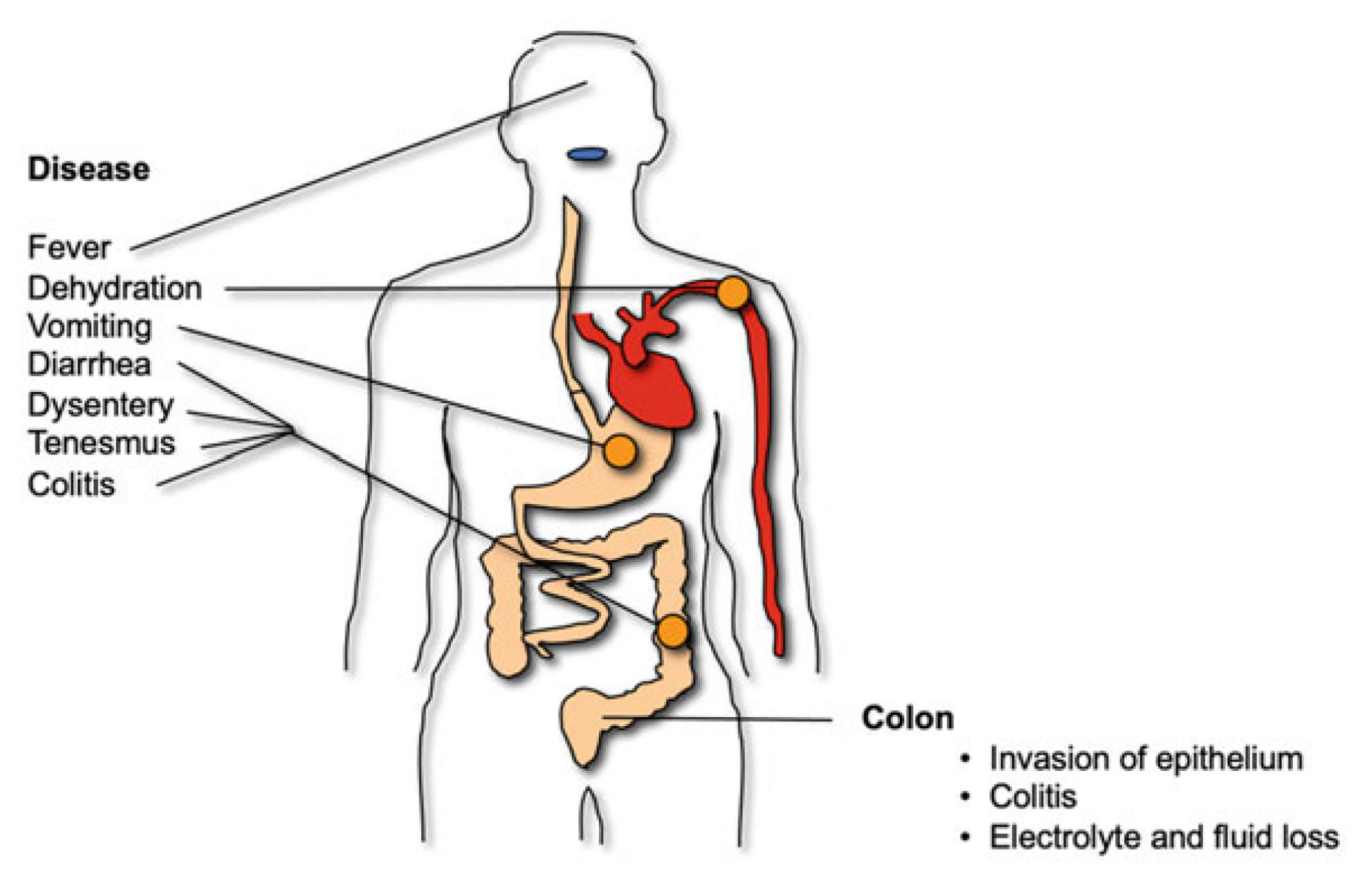
Clinical symptoms of infection with *Shigella* spp. Shigellosis is a self-limiting infection resulting in colonic epithelial lesions found in most patients, which is a consequence of innate and adaptive immune responses to infection. A hallmark of dysentery is small volume, bloody, mucoidal stools with a high number of neutrophils. Infection can also cause watery diarrhea, dehydration and contribute to malnutrition in endemic populations

The two *Shigella* strains currently available and characterized for use in CHIMs are 2457T, a *S. flexneri* 2a strain, and 53G, a *S. sonnei* strain (Text Box 1). Both of these challenge bacteria were wild-type antibiotic sensitive strains that have been well characterized and subsequently extensively evaluated in presumptively naïve subjects (DuPont et al. 1972b; Herrington et al. 1990; Kotloff et al. 1995a), as well as in studies of drugs (Taylor et al. 2006, 2008), passive immunoglobulins (Tacket et al. 1992) and novel vaccine candidates (Shaughnessy and Olsson 1946; DuPont et al. 1972b; Kotloff et al. 1995a; Coster et al. 1999; Pitisuttithum et al. 2016; Talaat et al. 2021) (Table 2). These bacteria, and their respective CHIMs, are a tremendous resource to the *Shigella* field, enabling detailed characterization of the clinical course of early disease that is not feasible in natural exposure, increasing the understanding of the host immune response to infection and the association between immune response and host susceptibility as well as aiding in the evaluation of multiple vaccines as promising candidates for further evaluation. Many of the operational aspects of the *Shigella*-CHIM are similar to those described for enterotoxigenic *Escherichia coli (*ETEC); the reader is referred to Fig. 1 of the ETEC-CHIM chapter for graphical representation of a CHIM study overview to supplement the information provided in this chapter on *Shigella*-CHIMs.

### 2.2 Nature of Challenge Agents and Delivery Systems

One of the most successful efforts to standardize the *Shigella* (and other enteric) CHIMs came with the realization that the cholera challenge was refined by pre-administering sodium bicarbonate prior to challenge and also by providing the inoculum in a similar buffer (Cash et al. 1974). While head-to-head studies of various buffers are lacking, *S. flexneri* 2a 2457T administered in sodium bicarbonate demonstrated a consistent and high shigellosis attack rate (AR) (Kotloff et al. 1995a). Further efforts to refine and/or standardize the model have included the evaluation of lyophilized and frozen challenge strains directly diluted and used for inoculation.

In the most exhaustive head-to-head comparison of various delivery platforms, freshly grown *S. sonnei* 53G administered in either sodium bicarbonate or milk, as well as an inoculum prepared directly from a frozen suspension, were compared (Kirkpatrick et al. 2000). Briefly, an initial cohort received 2000 colony forming units (CFU) of the frozen *S. sonnei* 53G challenge strain, which caused a mild atypical gastrointestinal illness in most participants; however, one subject experienced a severe, sepsis-like illness which led to the abandonment of the frozen inoculum as a challenge strategy. All subsequent groups received approximately 400 CFU of fresh, plate-grown bacteria in either milk or bicarbonate buffer. The use of sodium bicarbonate increased the AR to 71% from the 40% seen with milk. More recently, a lyophilized lot of S. *sonnei* 53G was evaluated at Cincinnati Children’s Hospital Medical Center (CCHMC) using increasing doses ranging from approximately 500 CFU to 1500 CFU in bicarbonate buffer with an AR of 40% in the highest dose cohort (1760 CFU) (Frenck et al. 2020). Although there are differences in ARs across these two studies, it is important to consider that different primary outcome definitions were used in each study with a much stricter definition employed in the CHIM conducted at CCHMC. When a less severe disease definition was applied to the high dose cohort of the CCHMC study, the AR increased to 70% (Frenck et al. 2020) (see Sect. 2.5 for additional discussion on disease endpoint definitions).

The value of the lyophilized challenge strain is that it removes the inherent variability of using freshly grown and harvested cells from plate scrapes for inoculation. Prior to the use of the lyophilized strain, trained microbiologists initiated a multi-day process of inoculum preparation that consisted of isolating virulent (Form I) *Shigella* colonies following overnight incubation of a challenge stock (Talaat et al. 2019). Virulent colonies were expanded and freshly harvested for inoculation introducing potential variability in colony selection, reagents, growth, etc. Minimizing variability (along with harmonizing clinical endpoints, discussed below) enables direct comparison of study data across researchers, institutions and over time.

### 2.3 Volunteer Selection and Screening

The current strategy for subject selection is focused on identifying a healthy subset of the adult population that is susceptible to shigellosis (Talaat et al. 2019) while maintaining the ability to enroll sufficient numbers of subjects in a clinical trial to achieve the desired outcome and generate results that are externally valid to a broader population. Subject selection relies on self-reported prior *Shigella* exposure, travel to *Shigella* endemic regions and/or occupational or other known exposure that would potentially reduce susceptibility to challenge. Serologic screening based on anti-LPS serum IgG ELISA titers has also been utilized with the intent to complement other inclusion/exclusion criteria and to identify immunologically naïve or infection susceptible subjects.

To develop the serological screening assay, serum from individuals clinically diagnosed with *Shigella* infection was assayed by ELISA and western blot for antibody reactivity directed to IpaB, IpaC and IpaD proteins. Similarly, serum from individuals with no prior history of exposure to *Shigella* was also assessed by ELISA and western blot. Individuals with prior exposure history and serum reactive with the Ipa proteins were designated as “true positives”, whereas individuals with no prior exposure history and serum with undetectable reactivity were designated as “true negatives”. Serum from these positive and negative populations was then assayed for anti-S. *flexneri* 2a LPS serum IgG responses. A titer of 2500 differentiated subjects in the two populations (Kaminski, personal communication). The 2500 anti-*S. flexneri* 2a LPS serum IgG titer was subsequently used as an exclusion threshold for participation in *S. flexneri* 2a CHIMs. The same exclusions titer of anti-*S. sonnei* LPS serum IgG was later applied to the *S. sonnei* CHIM, in deference to the absence of data on its relationship to antibody levels or clinical outcome with this serotype.

In a refinement of the *S. sonnei* 53G (lyophilized) model, the 2500 serological screening threshold titer excluded a small subset (3%) of potential participants and led to comparable baseline *S. sonnei* IgG titers across study groups (Clarkson et al. 2020). In the 53G challenged subjects, there was no subsequent association between the baseline serum IgG titer and the primary shigellosis endpoint. This is unlike the negative correlation observed in vaccinated subjects but not placebo subjects of the *S. flexneri* 2a CHIM (Talaat et al. 2021). Further immune response evaluations in the S. *sonnei* 53G refinement study revealed that baseline S. *sonnei* anti-LPS serum IgA titers were heterogeneous across study groups and subjects with higher serum IgA titers were less likely to develop shigellosis. Similar trends were observed in baseline fecal and memory B cell antibodies, with higher IgA responses differentiating subjects progressing to shigellosis from those who did not (Clarkson et al. 2020).

Analysis of serum IgG screening titers from all potential participants in the *S. sonnei* 53G model refinement study revealed that 70% of screened subjects had IgG titers ≤ 625, at least fourfold lower than the exclusion threshold. However, using the same screening method in a subsequent vaccination-challenge study using *S. sonnei* 53G (Frenck et al. 2021), the 2500 titer screening threshold allowed enrollment of a large proportion of participants with baseline LPS-specific antibody levels close to or above the IgG threshold reported as protective for *S. sonnei* in field studies (Cohen et al. 1988). Across all cohorts in the vaccination-challenge trial (Frenck et al. 2021), subjects with higher anti-*S. sonnei* IgG levels at baseline were significantly less likely to develop shigellosis (*p* = 0.003). Serum IgA titers are yet to be determined from this vaccination-challenge study but may also reveal additional important differences in immune responses associated with disease progression across cohorts in this study.

Additional studies are needed to determine if the 2500 serum IgG screening threshold established for *S. flexneri* 2a is appropriate for the *S. sonnei* CHIM. One could offer that the threshold established as protective in field trials (Cohen et al. 1988) could serve as an appropriate alternative; however, differences between ELISA methods need to be considered as they can contribute to a misalignment of titer determinations. However, an immune response that protects in a field setting may be insufficient in the experimental CHIM, which is optimized to promote a specific disease outcome (e.g., pre-challenge fasting, gastric acid buffering and controlled inoculum dose). This, in conjunction with the association of baseline IgA responses and differences in disease progression during model setup, highlights the potential need for expansion and/or modification of the serologic prescreening processes, at least for *S. sonnei* 53G. It is possible that serum IgG levels may not be the only (or the most) appropriate marker of immunity or resistance to infection. Alternative immune parameters and thresholds should be evaluated and established for use as screening tools in these (and new) CHIMs.

Furthermore, during the *S. sonnei* 53G (lyophilized) CHIM development, the use of a high baseline IgG threshold, which could equate with prior exposure to *S. sonnei* or cross-reactive antigens, may have resulted in the selection of an inoculum dose that was higher than what would be required in a truly susceptible population; thus, leading to a more stringent CHIM that would overcome vaccine-induced immunity. Collectively, these observations point to a few of the challenges when establishing new *Shigella*-CHIMs. It will be essential to confirm these observations in future studies and identify immune exclusion criteria specific for each CHIM in order to accurately complement other exclusion criteria and assist in the recruitment of a naïve population.

### 2.4 Dose-Ranging and Attack Rates

Across CHIMs, there has been an unclear association between inoculation dose and disease (Porter et al. 2013); however, this may have been due to variability in strain preparation and administration (selection of virulent colonies, pre-challenge administration of a buffer, administering the challenge in skim milk or sodium bicarbonate), subject selection (immunology screening) and/or outcome definitions. Supporting this theory are several studies in which various inoculum doses have been administered as part of the same study. For example, a two to threefold increase in the proportion of subjects meeting various clinical endpoints has been reported when the *S. flexneri* 2a 2457T dose was increased from 140 to 1400 CFU (Kotloff et al. 1995a). A similar increase in disease rates has also been seen when evaluating a range of *S. sonnei* 53G doses from 500 to 1500 CFU (Frenck et al. 2020). However, both observations are based on a limited sample size and therefore may benefit from expanded evaluation.

The clinical course of disease in both *Shigella*-CHIMs is as expected in an otherwise healthy, immunocompetent adult with shigellosis and is characterized by gastrointestinal symptoms of diarrhea, dysentery, vomiting, nausea, anorexia and abdominal pain, and/or cramps. Additionally, systemic symptoms of fever, malaise, headache, myalgia and arthralgia are common (Fig. 1). While there are differences in the constellation of symptoms and their severity between the two evaluated strains, the proportion of exposed subjects experiencing each individual symptom is comparable (Table 3). Interestingly, fever and anorexia appear to be more prevalent in subjects challenged with S. *flexneri* 2a 2457T while dysentery appears to be more common in subjects challenged with *S. sonnei* 53G. It is important to note that while these potential differences are intriguing, no studies have been performed to date where the clinical profile of shigellosis induced by the two challenge strains has been studied simultaneously under the same clinical research protocol.

### 2.5 Common Disease Endpoints Are Used Across Both Established Shigella-CHIMs

A critical aspect of CHIMs is the choice of endpoints and case definitions, as these have significant ramifications on the study results and consequently affect decision-making. Endpoints may represent a variety of disease/infection stages including carriage, shedding, infection (i.e., detection of the pathogen), clinically overt disease and its severity, including the extent of intestinal and systemic inflammation. While the choice of disease endpoints is specific to the infection of interest, endpoints are also required to be aligned with the target product profiles in order to assess intervention effects. Generally, endpoints that estimate vaccine efficacy against the most relevant aspect of the disease from a public health perspective should be deemed more relevant than others.

One of the complicating aspects of comparing the signs and symptoms of shigellosis over time and among institutions has been the inconsistency in primary endpoint definitions (Porter et al. 2019). For example, fever, diarrhea and dysentery have each been defined at least in six different ways in previously published *Shigella*-CHIMs and each has been included individually and collectively as components of a primary endpoint (Porter et al. 2019).

Recently, investigators representing multiple institutions utilizing *Shigella*-CHIMs gathered to evaluate previously utilized primary endpoint definitions and to evaluate a potentially harmonized primary endpoint that could be the standard for use in subsequent CHIMs (Text Box 2) (MacLennan et al. 2019b). One of the key elements in developing a consensus endpoint was to balance the AR with a clinically meaningful endpoint. For example, while a very high proportion of subjects develop some level of illness following the experimental *Shigella* challenge, some illnesses can be quite mild. While including overly mild illnesses as a primary endpoint may increase the AR, and it may also yield a model that does not represent the typical clinical picture of more severe disease, which in turn may decrease the ability to appropriately evaluate vaccine efficacy. In contrast, a model that has a too severe clinical endpoint may yield very low ARs requiring large sample sizes for sufficiently powered vaccine efficacy studies. Balancing these aspects, the investigators finalized a primary endpoint that comprised elements of diarrhea, fever, dysentery and other constitutional symptoms as a consensus shigellosis definition for future use in the currently established *Shigella*-CHIMs (MacLennan et al. 2019b).

### 2.6 Disease Endpoints Versus Severity Scores

Utilizing individual subject-level data from multiple *S. flexneri* 2a CHIMs, the co-occurrence and severity of the numerous signs and symptoms of shigellosis were evaluated (Porter et al. 2018). These analyses were used to develop a disease severity score to provide a more granular characterization of the disease-induced across the disease complex range. While the score was established using only data from *S. flexneri* 2a studies, it appeared to be equally applicable to disease following challenge with *S. sonnei* given the reported correlation of increasing disease severity score with increasing challenge dose of *S. sonnei* 53G (Frenck et al. 2020).

In addition to enabling a more robust characterization of the shigellosis disease complex in the *Shigella*-CHIM, the disease severity score offers an alternative endpoint for consideration in the assessment of candidate vaccines (Fedorov et al. 2009). Briefly, dichotomous or nominal endpoints are less statistically efficient than endpoints that are ordinal (or continuous). Thus, the use of the disease severity score in comparing disease between naïve and vaccinated subjects is likely to provide more statistical power to differentiate the disease across the two groups than what is afforded with a dichotomous endpoint such as shigellosis. The utility of the disease severity score has been demonstrated in evaluating the effect of a *Shigella* bioconjugate vaccine in reducing the severity of illness induced by *S. flexneri* 2a 2457T in a vaccination-challenge study (Talaat et al. 2021). The vaccine’s greatest effect appeared to be on reducing the severity of illness following the challenge. While the proportion of vaccine and placebo recipients meeting the a priori dichotomous shigellosis endpoint was not significantly different, vaccinated subjects had statistically significant lower disease severity compared to placebo subjects. Particularly interesting, the most striking difference in disease severity across treatment groups was among participants that met the a priori primary endpoint, highlighting the importance of improved granularity in characterizing shigellosis and disease outcomes in the CHIM.

The disease severity score may also provide a unique endpoint for the comparison of non-clinical responses, including immunologic outcomes (Talaat et al. 2021; Clarkson et al. 2020). Following challenge with *S. sonnei* 53G, a significant correlation between multiple post-challenge adaptive immune responses and the disease severity score was observed (Clarkson et al. 2020). Additionally, novel evaluations of fecal markers of intestinal inflammation post-challenge with *S. sonnei* 53G revealed a significant association with increasing intestinal inflammation and disease severity score, indicating that the severity of the disease may also be directly linked with the innate immune response. Similar associations of adaptive immune responses and disease severity score have also been reported in an *S. flexneri* 2a CHIM study where, following vaccination with a *Shigella* bioconjugate vaccine, pre-challenge anti-LPS serologic responses were significantly inversely correlated with disease severity score following challenge with *S. flexneri* 2a 2457T (Talaat et al. 2021). The *Shigella* disease severity score synthesizes multiple clinical signs and symptoms into a single endpoint, enabling a robust comparison across treatment groups; it also appears to correlate with non-clinical outcomes, potentially highlighting differences not readily apparent using traditional endpoints.

## 3 The Role of CHIMs in Shigella-Specific Immune Response Investigations

### 3.1 Shigella-Specific Immunity and Key Antigens

Homologous re-challenge studies in the same volunteers offer the opportunity to study *Shigella* serotype-specific immunity. Three separate challenge–re-challenge studies have demonstrated the ability of an initial experimental infection to induce a sufficiently robust immune response to protect against re-challenge with the same organism (DuPont et al. 1972b; Herrington et al. 1990; Kotloff et al. 1995a). In a seminal study of homologous protection, of the subjects previously challenged with *S. flexneri* 2a 2457T who developed shigellosis (n = 15), only 20% developed fever or diarrhea following re-challenge with 10,000 CFU of *S. flexneri* 2a 2457T (DuPont et al. 1972b). In contrast, naïve participants (n = 39) developed fever or diarrhea at a much higher proportion (56%). Interestingly, there was no difference in the proportion of subjects shedding the challenge strain in their stools across the two groups (67% and 69%). Following on from this work, the inoculation solution was changed from skim milk to sodium bicarbonate and a more consistent AR was achieved with additional gastric buffering. In the same study, among veterans previously challenged with 1000 CFU of 2457T (n = 5), when re-challenged with the same strain, subjects shed lower amounts of *Shigella* and only 40% experienced any illness while 20% had fever or dysentery and none had diarrhea (Kotloff et al. 1995a). In contrast, among naïve subjects (n = 12), 92% had some illness with 83% having either fever, diarrhea or dysentery.

There is a single published challenge–re-challenge study with *S. sonnei* 53G (400 CFU). Among the 12 naïve participants, 50% had fever, 58% diarrhea and 67% dysentery, while only 1 of 6 re-challenged participants developed dysentery (a single dysenteric stool) (Herrington et al. 1990). These challenge–re-challenge studies, in conjunction with epidemiological studies (DuPont et al. 1972b; Ferreccio et al. 1991; Formal et al. 1991), have been instrumental in demonstrating the role of serotype-specific immunity in protection from shigellosis. Although the exact mechanism of protection is unknown, the observed homotypic protection has solidified the OAg as a key protective antigen leading to the generation of serotype-specific OAg-based immune responses as the focus of most *Shigella* vaccines under development (Table 1).

Nonetheless, additional *Shigella* antigens, including highly conserved Ipa proteins as well as the essential virulence factors/antigens VirG (IscA) and PSSP-1, have also been associated with the development of protective immunity and may be the targets for vaccine development (Clarkson et al. 2021a, b; Shimanovich et al. 2017; Ndungo et al. 2018). Recently, a novel *Shigella* proteomic array was utilized to evaluate serum samples from subjects receiving the *S. flexneri* 2a 2457T challenge strain. Data indicated a strong association between antibody responses to IpaA, IpaB and IpaC and protection from shigellosis upon challenge (Ndungo et al. 2018). In a similar application of the proteome array using samples collected from the *S. sonnei* CHIM dose-finding/dose-verification study (Frenck et al. 2020; Clarkson et al. 2020), subjects that were resistant to infection had elevated antibodies directed to LPS and IpaB (Randall 2021) indicating that anti-Ipa antibodies may complement anti-LPS antibodies and further reduce the risk of developing shigellosis following challenge (Clarkson et al. 2021a; Shimanovich et al. 2017; Ndungo et al. 2018).

### 3.2 Potential for Cross-Protection Across Shigella Serotypes

Given the number of *Shigella* serotypes, investigating antigens that may provide cross-protection across multiple serotypes is of high interest. Some vaccine strategies have focused only on the highly conserved Ipa proteins in hopes of inducing pan protection from all *Shigella* species (Martinez-Becerra et al. 2012, 2013). Their potential value as vaccine antigens is further supported by animal studies that indicate induction of protective OAg-independent immunity (Martinez-Becerra et al. 2012, 2013). However, given the extensive evidence that protection from *Shigella* infection is serotype-specific, it is most likely that the OAg will be a required component of a protective vaccine. Nonetheless, given the homology of these protein antigens across *Shigella* serotypes, it is possible that robust immune responses to these highly conserved Ipa proteins may provide additional breadth of immunity and could potentially provide protection across heterologous serotypes (at least within a given serogroup) (Noriega et al. 1999).

The potential for cross-protection may be a more realistic goal within certain serogroups of *Shigella* species. For example, there are structural similarities between the LPS OAg repeating units of *S. flexneri* serogroups with the potential for cross-reactivity between some of the serotypes and subserotypes. The overlapping presence of several type- and group-specific antigens on the respective OAg structures is the foundation for OAg-based vaccine strategies targeting cross-protection across multiple *S. flexneri* serotypes. For example, the OAg portion of LPS of *S. flexneri* 2a (representing group factor 3,4 and type factor II) and 3a (representing group factors 6 and 7,8 and type factor III) could theoretically provide protection against all known serotypes of *S. flexneri*, except serotype 6, which expresses a different linear tetrasaccharide repeating unit of LPS. This potential of cross-protection was demonstrated in guinea pigs immunized with live-attenuated *S. flexneri* 2a and 3a vaccine candidates, leading to the generation of cross-reactive serum and mucosal antibody responses against all *S. flexneri* serotypes, except for *S. flexneri* 6. When these immunized guinea pigs were challenged with virulent wild-type *S. flexneri* strains, vaccination provided significant cross-protection against serotypes 1b, 2b, 5b, and Y, but not serotypes 1a, 4b, or (as anticipated) 6 (Noriega et al. 1999).

In a field trial of *S. flexneri* 2a LPS-conjugate vaccine in 1–4-year-old Israeli children, * 52% efficacy against shigellosis caused by S. *flexneri* serotype 6 was observed (Passwell et al. 2010). Further investigation of the serological responses from field trials of the *S. flexneri* 2a conjugate vaccine demonstrated some cross-reactivity with *S. flexneri* 6, but serum responses from natural infection with *S. flexneri* 2a did not cross-react against serotype 6 (Farzam et al. 2017). The shared *O*-acetylated disaccharide of these two serotypes could be the explanation for this observed cross-reactivity.

The existing *Shigella* CHIMs have supported the development of single serotype *Shigella* vaccines by providing an early indication of homotypic clinical protection. The existing and future *Shigella* CHIMs will also provide valuable assessment of multi-serotype *Shigella* vaccine candidates, including an early indication of both homotypic and heterotypic protection resulting from immunologic cross reactivity.

### 3.3 Immune Correlates of Protection for Shigella Infection

**Fig. 2:**
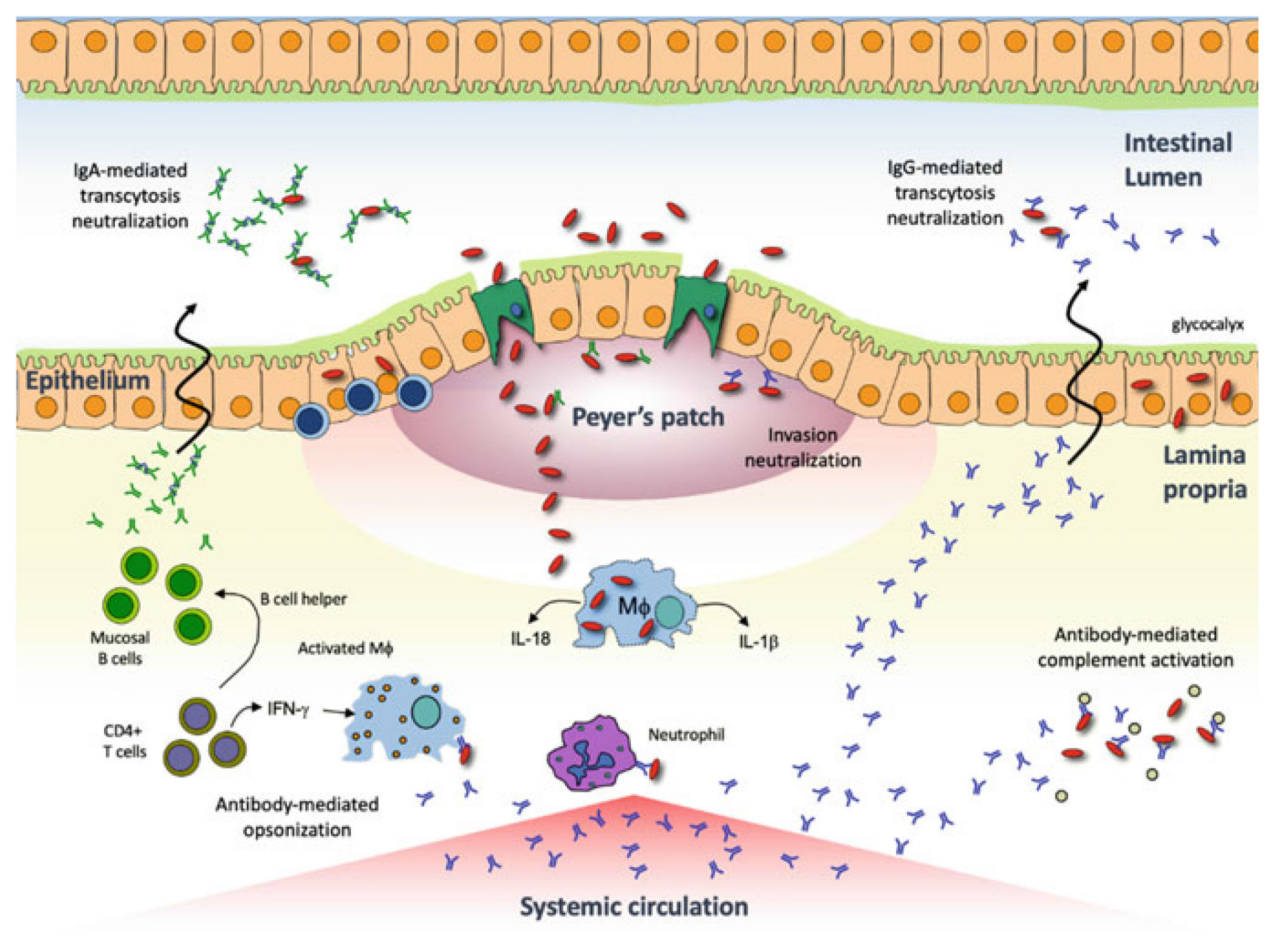
*Shigella* pathogenesis and potential protective immune mechanisms. *Shigella* is transmitted via the fecal-oral route and transits from the lumen into the lamina propria via M cells. Once in the basolateral space, *Shigellae* can induce pyroptosis in macrophages (MΦ) and utilize a type-III secretion system to translocate effector proteins (i.e., Ipa proteins) resulting in phagocytosis of the bacterium. Once intracellular, *Shigellae* multiply and spread to adjacent cells by reorganizing host cell actin polymerization. The intercellular bacterial spread and subsequent recruitment of neutrophils to the site of infection results in tissue destruction and the characteristic pathology of shigellosis. Although well-defined correlates of immunity have not been fully elucidated, protective immune responses may include neutralization of the bacteria in the gut lumen (prevention of M cell transit) or in the lamina propria (interference with host cell invasion), antibody-mediated complement activation (bacterial killing) and opsonization (marking bacteria for phagocytosis)

When considering *Shigella* pathogenesis, several protective immune mechanisms could be proposed depending on the clinical endpoint of interest (Fig. 2). If sterilizing immunity and prevention of transcytosis across the mucosal epithelium is desired, antibodies actively secreted locally or passively transudated into the lumen may be required. Protection from mild to moderate disease could be achieved within the intestinal lamina propria via antibody-mediated neutralization, killing by opsonization or complement activation or, antibody-dependent cellular cytotoxicity. Cell-mediated immunity may be important in protecting from more severe disease by working to prevent or reduce the intercellular spread of *Shigellae* and subsequently reducing the frequency or severity of bloody, mucoidal stools and colonic ulceration.

An immune CoP for *Shigella* infection has yet to be defined. LPS-specific serum IgG has been suggested as a CoP (Talaat et al. 2021; Passwell et al. 2001, 2010; Cohen et al. 1991, 2019; Robbins et al. 1992); however, it is yet to be defined if LPS-specific serum IgG is serving as a mechanistic correlate or as a surrogate measure for a yet-to-be-defined protective mechanism. An *S. sonnei* LPS-specific serum IgG threshold was previously established as protective in field trials (Cohen et al. 1988). However, in a recent vaccination-challenge study, comparable or higher anti-LPS IgG levels were achieved by nearly 70% of subjects yet were insufficient to prevent shigellosis following *S. sonnei* challenge (Frenck et al. 2021). Nonetheless, baseline and pre-challenge anti-LPS IgG levels were higher in subjects who did not develop shigellosis post-challenge, thus suggesting a role of these antibodies in clinical protection.

Additional CoP have also been suggested, including LPS-specific serum IgA and functional antibody responses (Clarkson et al. 2021a, 2020; Shimanovich et al. 2017), and several different protective mechanisms have been proposed as a result of data generated in *Shigella*-CHIMs. These infection models have demonstrated the importance of LPS-specific mucosal antibody responses, as measured either by mucosal IgA secreting B cells or by quantifying secretory IgA antibodies in mucosal secretions (Holmgren et al. 2017; Clarkson et al. 2021a, 2020; Coster et al. 1999; Schultsz et al. 1992; Cohen et al. 1996). Additionally, both LPS- and IpaB-specific memory B cell IgA responses have been associated with protective efficacy or a reduced risk of disease post-challenge in a CHIM (Clarkson et al. 2021a, 2020; Wahid et al. 2013). Although T cell immunity has been extensively investigated in the context of *Shigella* pathogenesis and immune evasion, there has been little evidence derived from CHIMs to suggest their utility as CoP for *Shigella* infection (Holmgren et al. 2017). Mathematical modeling or machine learning studies have described a combination of LPS-specific mucosal (IgA antibody secreting cells) and systemic (serum IgG) responses working synergistically together serving as co-correlates (Davis et al. 2013; Arevalillo et al. 2017).

Recently, anti-LPS IgG and serum bactericidal responses following vaccination with an *S. flexneri* 2a bioconjugate vaccine were associated with protection following challenge with *S. flexneri* 2a strain 2457T (Clarkson et al. 2021a). Using extensive methods to characterize the immune responses to vaccination and challenge in a controlled environment as afforded by the CHIM may offer an opportunity to differentiate a protective immune response that may otherwise be indiscernible in field studies. Similarly, placebo subjects challenged with either *S. sonnei* (Clarkson et al. 2020) or S. *flexneri* 2a (Clarkson et al. 2021a) can be compared and contrasted enabling dissection of potential differences across serotypes that may be essential for protective immunity.

### 3.4 Shigella-Specific Immune Profiles

Defining immune CoP can be complicated due to a variety of host and environmental factors (Katona and Katona-Apte 2008; Flores and Okhuysen 2009; Mondal et al. 2012; Zimmermann and Curtis 2019). Additionally, pathogen strain or serotype, route of infection and dose can influence the host’s immune response and pathogen virulence (Martins et al. 2013; Sela et al. 2018; Sperandio 2018). Furthermore, a protective immune response for *Shigella* infection may actually be composed of several highly correlated responses across a range of immunoassays measuring multiple different systemic, mucosal or cellular responses (Clarkson et al. 2021b). Consequently, attempts to define a single immune correlate or surrogate of protection may not adequately account for all of the described complexities and a broader definition of protective immunity describing an integrated and networked immune response profile should be considered (Clarkson et al. 2021b; Querec et al. 2009; Pulendran et al. 2010; Trautmann and Sekaly 2011; Li et al. 2013; Nakaya and Pulendran 2015; Haks et al. 2017; Hasin et al. 2017). Immune response profiles post-vaccination and/or challenge or across different vaccine constructs or exposure routes have been described for several pathogens including human immunodeficiency virus (HIV), polio, *Salmonella* Typhi and cholera (Querec et al. 2009; Ryan and Calderwood 2000; Plotkin 2001, 2010; Tomaras and Plotkin 2017; Alter and Barouch 2018; Dahora et al. 2019).

Recent data from two *Shigella*-CHIMs have described distinct protective immune profiles associated with different antigenic exposure routes: parenteral immunization with the *S. flexneri* 2a bioconjugate and oral challenge with either *S. flexneri* 2a 2457T or *S. sonnei* 53G. The protective immune profile associated with parenteral immunization was characterized by robust systemic IgG and IgA responses with milder functional antibody activity and mucosal IgG responses (Clarkson et al. 2021a, 2021b). As expected, a different immune profile was observed after oral challenge with S. *flexneri* 2a that induced a more “balanced” profile characterized by similar relative increases in both systemic and mucosal antibody responses with moderate memory immune responses and functional antibody activity. Surprisingly, an altogether different immune profile was observed after oral challenge with *S. sonnei* characterized by robust increases in mucosal IgG and IgA responses, clearly differentiating subjects without shigellosis from those that displayed clinical signs post-challenge (Clarkson et al. 2021b, 2020), while little to no differences in systemic responses were observed across these subjects. Data from these CHIMs suggest that: (1) different immune profiles can provide protection against *Shigella* infection and (2) different protective mechanisms may be associated with protection from different *Shigella* species. CHIMs provide a valuable tool to conduct in-depth immune response characterization and to investigate and describe early immune profiles associated with protection from shigellosis. Interestingly, the immune profile differences observed between *S. flexneri* 2a and *S. sonnei* are also consistent with the differences in pathogenesis between these two serotypes (see Sects. 1.2 and 1.3).

### 3.5 Vaccine Immunogenicity and Effectiveness in Target Age Group—Cautionary for CHIMs

A limited number of *Shigella* vaccines have been evaluated in children or infants living in LMICs. An orally delivered live-attenuated *S. sonnei* vaccine in Bangladeshi children aged 5–9 years lacked immunogenicity post-vaccination (Raqib et al. 2019). There was also no shedding of the vaccine strain among immunized children, indicating that the attenuated bacteria were unable to colonize the intestinal mucosa. Resistance to colonization is not uncommon with oral vaccines in endemic populations and has been reported with other enteric pathogens, including cholera and rotavirus (Qadri et al. 2005, 2007; Madhi et al. 2010). Although many factors likely contribute to a lack of vaccine colonization, consideration must be given to the high incidence of EE that exists in the target population. Environmental enteropathy significantly impacts proper gut immune function and intestinal permeability, contributing to a constant state of malnutrition combined with severe micronutrient deficiencies (Korpe and Petri 2012).

The impact of EE should also be specifically considered in the context of *S. sonnei* infection and *S. sonnei* live-attenuated vaccine strains given recent evidence that *S. sonnei* harbors a T6SS and uses it to kill commensal intestinal bacteria (Anderson et al. 2017). The importance of commensal bacteria has been well documented as they not only contribute to proper gut health but have also been shown to influence and modulate systemic immune responses to pathogenic bacteria (Molloy et al. 2012; Belkaid and Hand 2014). With S. *sonnei* infections increasing in prevalence in LMIC populations (Rogawski et al. 2018; Thompson et al. 2015; Obiero et al. 2017; Pavlinac et al. 2017) and, with the testing of live-attenuated *S. sonnei* vaccine strains, it is essential to consider how this serotype may exacerbate EE and further contribute to reduced intestinal barrier function and increased malnutrition in the target population. Furthermore, the killing of intestinal microbiota may increase the possible risk of systemic *Shigella* bacteremia, leading to serious health complications or death in an already susceptible population.

*S. sonnei* and *S. flexneri* 2a OAg-conjugate vaccines have also been studied in the target population and have been shown to be safe and immunogenic in Israeli children as young as 1-year-old (Passwell et al. 2010, 2003; Ashkenazi et al. 1999). The effectiveness of the *S. sonnei* and *S. flexneri* 2a OAg conjugates has also been evaluated with promising reports of * 70% efficacy in Israeli adults. The effectiveness of these candidate conjugate vaccines was also tested in 1–4-year-old Israeli children; however, the efficacy results were age-related with only children ≥ 3 years old protected from shigellosis (Passwell et al. 2010). This age-related efficacy in a *Shigella* endemic region, where the majority of infections occur prior to the age of three years, suggests that the observed efficacy in older populations may have partially resulted from the conjugate vaccine serving as a booster to previously exposed, and therefore mucosally primed, individuals.

*Shigella*-CHIMs are useful tools for preliminary readouts of vaccine efficacy; however, without defined CoP for *Shigella* infection, caution is required when extrapolating efficacy results from CHIMs to the target population. It is important to consider that CHIM participants are adults, typically living in high-income settings, and are distinctly different than the target population of young children living in LMICs. Infant health, nutritional status and immune status can vary greatly in endemic settings, all of which contribute to differences in immune and vaccine responses (Mondal et al. 2012; Zimmermann and Curtis 2019). Furthermore, the immune system does not fully mature until approximately 24 months of age and infants have been shown to have lower levels of circulating immunoglobulin and complement effectors (Simon et al. 2015), with a recent report indicating a gradual increase in both *Shigella*-specific serum IgG and IgA responses in infants over time (Chisenga et al. 2021). This is an important consideration in the context of the currently proposed CoP for *Shigella* infection, including serum IgG or functional antibody activity (Shimanovich et al. 2017; Cohen et al. 2019; Ndungo and Pasetti 2020). It is possible that protective mechanisms observed in one population may not extrapolate to all populations and that different protective immune mechanisms may be required depending on the population of interest.

## 4 Alternatives to *Shigella-CHIMS*

Animal models and cell culture systems using immortalized human cell lines or human tissue explants have historically been used to advance our understanding of the molecular and biochemical basis of the pathogenesis and immunology of enteric bacterial pathogens, like *Shigella* (Wenzel et al. 2017). However, it has long been recognized that these models have shortcomings that limit their translational applications, especially in drug and vaccine development. With the high burden of acute and long-term illness associated with *Shigella*, as well as the alarming rise in antibiotic resistance, the need to accelerate drug and vaccine development has become more urgent and, in addition to CHIMs, improved in vitro models are needed.

### 4.1 Commonly Used Animal Models

Several Shigella-specific animal models have been developed (Kim et al. 2013). For vaccine efficacy studies, two pre-clinical models have been utilized extensively. The mouse pulmonary lung model has been utilized by several groups to demonstrate preliminary immunogenicity and efficacy, largely as a stepping stone to testing in higher animal species (Mallett et al. 1993). The guinea pig keratocon-junctivitis or Sereny model has been utilized to evaluate immunogenicity and efficacy (Hartman et al. 1991). Both models rely on the protection of mucosal surfaces, albeit dissimilar to the intestinal tract and at bacterial doses that exceed those utilized in CHIMs. Although some evaluations have determined that mucosal antibody (ocular IgA) correlates with protection from disease (Kaminski et al. 2014), transferability to the human model has not been established.

A guinea pig rectocolitis model has been described (Shim et al. 2007) and with some slight technical modifications has been utilized successfully in vaccine efficacy studies; however, its use is not widespread. The advantage of the guinea pig rectocolitis model is that infection closely replicates human disease with bloody mucoidal stools, tenesmus and intestinal inflammation. More recently, a mouse diarrhea model has been developed by investigators at the University of Virginia that utilizes mice maintained on a zinc-deficient diet (Medeiros et al. 2019). This promising diarrhea/dysentery model is potentially a significant advancement because it also mirrors the human clinical outcomes resulting from natural or experimental *Shigella* infection (Shim et al. 2007; Medeiros et al. 2019; Barry et al. 2019). Both the guinea pig rectocolitis and mouse zinc-deficient models may also enable investigators to evaluate vaccine impact on other negative health outcomes from *Shigella* infection, e.g., gut inflammation and growth impairment (Medeiros et al. 2019, 2020). The new zinc-deficient mouse model has been used successfully to evaluate both active (vaccine) and passive (monoclonal antibody, mAb) interventions for the prevention of shigellosis (Medeiros et al. 2020; Teh et al. 2021).

In addition to small animal models, two non-human primate models have been utilized to evaluate potential *Shigella* countermeasures. The rhesus macaque model has proven extremely useful in the identification of *Shigella* antigens recognized by the immune system after oral infection (Oaks et al. 1986, 1996; Turbyfill et al. 1995) and establishing that prior infection with one *Shigella* serotype offers resistance to infection with the homologous serotype but not heterologous serotypes (Formal et al. 1991). Rhesus macaques have also been used in early efficacy studies of promising *Shigella* vaccine candidates (Formal et al. 1965, 1984). More recently, an *Aotus nancymaae* model was established and utilized to evaluate *Shigella* vaccine candidates (Gregory et al. 2014). The *Aotus* model has the added advantage of being capable of potentially evaluating combination enteric vaccines as the model has also been established for both enterotoxigenic *Escherichia coli* (ETEC) (Jones et al. 2006a; Rollenhagen et al. 2019) and *Campylobacter* spp. (Jones et al. 2006b).

However, the benefits of both non-human primate models must be considered against several inherent constraints, which include the requirement for a high inoculum dose to achieve reproducible attack rates and the development of gastric lesions, which are neither associated with human shigellosis nor evident in CHIMs. Additionally, it is unclear if protection seen in these models directly correlates to human efficacy (in CHIM studies or field efficacy trials). Costs and ethical considerations also must be factored into the decision matrix, which can reduce the attractiveness and value of these non-human primate models for vaccine efficacy assessments.

### 4.2 Human Enteroid Model

Over the last 10 years, the human enteroid model has rapidly evolved to become a more human-relevant research tool, with the potential to be transformational in drug and vaccine development (Ranganathan et al. 2020; Foulke-Abel et al. 2020). The full research potential of this ex vivo model is yet to be realized but early application to *Shigella* indicates that it is suitable for studies of pathogenesis and early events such as colonization, invasion and activation of innate immunity, as well as triggers of gut inflammation (Ranganathan et al. 2019; Koestler et al. 2019). The model has recently shown that exposure of *S. flexneri* 2a to glucose and bile salts upregulates the expression of multiple adhesins suggesting that *Shigellae,* as highly human-adapted pathogens, have evolved to regulate virulence gene expression for efficient colonization and infection of the human host (Chanin et al. 2019). This highlights the importance of this new enteroid model for studies of bacterial enteric pathogens and, in the case of *Shigella*, the identification of new adherence factors as novel targets for future vaccine development efforts and the exploration of their role in facilitating infection in future CHIMs with *S. flexneri* and *S. sonnei* strains. The encouraging early enteroid model results of *Shigella* pathogenesis complement observations made in the CHIMs (Bourgeois, personal communication) and also suggest that further modification of the enteroid model, using cells derived from *Shigella* immune individuals, CHIM-infected individuals or individuals immunized with leading vaccine candidates, should be pursued to better understand bacterial and host cell interactions.

## 5 Next Steps in *Shigella-CHIMs:* Expanding the Footprint

### 5.1 Extending Established Models to Endemic Populations and Regions

The conduct of CHIMs, including those for *Shigella,* has expanded into individuals living in endemic areas (Bodhidatta et al. 2012). This has largely been the result of increased funding for these studies in endemic populations and regions. Recent international meetings sponsored by the Wellcome Trust, International Alliance for Biological Standardisation (IABS) (Pollard et al. 2020), WHO and the Gates Foundation have all recognized that CHIMs performed in endemic populations would differ from those in non-endemic settings. Potential distinctions may be due to differences in pathogen exposure and acquisition of naturally acquired immunity, host genetic background, gut microbiome and co-morbidities. As a result, the findings from CHIMs undertaken in non-endemic populations may not be generalizable (WHO 2016; Gordon et al. 2017; Elliott et al. 2018; Baay et al. 2019; Kunda-Ng’andu et al. 2021). Establishing CHIMs in endemic settings requires close collaboration between laboratories and clinical sites that have well-established CHIMs in order to benefit from previous experience, allowing for model transfer, as well as proactive dialogues with regulatory and ethical agencies of endemic countries (Kunda-Ng’ andu et al. 2021).

Key attributes of endemic CHIMs would be the ability to evaluate disease susceptibility or severity known to be impacted by population diversity or genetics (e.g., prevalence of sickle cell anemia in African populations, or lower neutrophil counts in persons of African origin and development of benign neutropenia following potent immune stimulation, etc.). Particularly for *Shigella***-CHIM** outcomes, pre-existing immunity, co-infections, exposure to cross-reacting pathogens, co-morbidities or infectious disease history, diet and enteric microbiome may alter responses by skewing the subject’s immune response pattern. Thus, CHIMs in endemic settings can confirm critical safety and end-point assessments of the model and as well aid in establishing early insight into the efficacy and possibly CoP in the target population, albeit perhaps not in the target age group.

Limitations for CHIMs are similar when carried out in unexposed populations and endemic settings (differences between natural and experimental infection, inoculum sizes, challenge strains, lack of accurate patient medical history, etc.). CHIMs have been conducted in LMICs since the 1990s involving cholera, rotavirus, malaria, *Shigella*, pneumococcus and dengue, thus allowing local capacity building and local investigators to play a pivotal role in the development of novel disease interventions and control measures that are relevant to their settings.

Transition of a CHIM to an area in which the pathogen is endemic requires additional development to re-assess parameters of the model including safety, clinical endpoints, infective doses and intervention efficacy in a potentially pre-exposed population. Typically, individuals repeatedly exposed to a pathogen over time will acquire immunologic responses that can impact disease susceptibility and/or resistance. Natural immunity is reflected by modified disease characteristics, morbidity and mortality compared to a naïve population, with profound impact on observed drug treatment or vaccine efficacy (Zimmermann and Curtis 2019). Level of prior exposure, and hence baseline immunity (Cohen et al. 1991, 1989, 1992; Obiero et al. 2017; Raqib et al. 2002), may influence the challenge dose needed to replicate a similar AR or illness compared to those observed in naïve population CHIMs. However, this may change the safety profile of the challenge. Alternatively, CHIMs in endemic populations provide an excellent opportunity for the identification of immunological signatures that would improve our understanding of host–pathogen and/or host–vaccine interactions and the spectrum of inflammatory and antigen-specific humoral and cellular responses associated with infection, immunity and protection in the context of naturally acquired immunity and historic exposure (Table 4). Findings in adult populations from endemic settings also need to be compared to findings in naïve adult populations, as for many prophylactic products protecting the individual, naïve or exposed, is a common goal.

### 5.2 Learnings from Conduct of Shigella-CHIMs in Endemic Settings

To date, there have only been two *Shigella*-CHIM studies in an endemic country, both in Thailand. The studies were conducted among healthy Thai adults at Mahidol University first as an infectivity dose-finding study (Bodhidatta et al. 2012) and then as a vaccine efficacy study (Pitisuttithum et al. 2016). In an attempt to recruit an infection-susceptible population from within the *Shigella* endemic setting, a more stringent screening threshold was applied to the Thai population compared to studies conducted in North America. In the dose-finding study (Bodhidatta et al. 2012), 3 cohorts of 12 subjects each were challenged with *S. sonnei* 53G strain (frozen). Volunteers with a baseline anti-S. *sonnei* LPS IgG > 1:800 (20% of those screened) were excluded to minimize the impact of pre-existing antibody titers from interfering with the infectivity. The 1:800 threshold was chosen (Dilara Islam, personal communication), based on previous field trial data collected in Israel (Cohen et al. 1989); however, as outlined earlier, different screening thresholds may be required in different CHIM settings, even across different *Shigella* endemic areas. Target inocula were 100, 400 and 1600 CFU, which were achieved for each of the cohorts. While the majority of volunteers in each group shed the challenge strain, 41–75% of the volunteers had at least one episode of dysentery, and only 1 volunteer in the highest dose cohort met the pre-specified criteria for shigellosis (diarrhea and/or dysentery with fever, ≥ 1 severe intestinal symptom and shedding). A single volunteer in each of the mid- and high-challenge doses met the definition of diarrhea. At the highest challenge dose, 75% of the Thai volunteers met the primary disease endpoint (diarrhea, dysentery and/or fever).

To achieve the desired AR, the target 1600 CFU dose was higher in Thai volunteers than previously used in immunologically naïve volunteers. In HICs, the S. *sonnei* 53G (frozen) challenge strain delivered at 400–500 CFU was able to induce shigellosis in 5/12, 6/11 or 5/9 controls (approximately 50%) in three independent studies (Herrington et al. 1990; Munoz et al. 1995; Black et al. 1987). The need for a greater inoculum in the Thai study, as compared to that used in North America, may be due to the underlying immunity in the Thai population despite screening for *S. sonnei*-specific anti-LPS antibodies. Given the small cohort size, the investigators were unable to determine if baseline antibody responses correlated with infection or clinical disease.

In a follow-up vaccine efficacy challenge study in Thailand evaluating the live-attenuated *S. sonnei* WRSS1 vaccine, the investigators were unable to reproduce the 75% AR observed in the dose-finding study at the highest challenge dose (Bodhidatta et al. 2012) as only a 20% AR was achieved (Clarkson et al. 2020). All volunteers in this study were screened for anti-*S. sonnei* LPS antibodies (1:800 threshold) and 24% were excluded from participation due to high baseline antibody titers; this was comparable with the previous study. Interestingly, a key finding of the second study was that anti-LPS IgA titers were identified as associated with protection. This finding has since been corroborated in *S. sonnei* 53G (lyophilized) challenge infectivity study in naïve populations in the US (Clarkson et al. 2020). However, it is important to note that the challenge inoculum using traditional methods of preparation by plate scrapes was identified as not being reproducible and could have accounted for the low AR and findings of low vaccine efficacy in Thai adults. Additionally, differences in endpoint definitions and other factors unique to the endemic Thai population that were not investigated may account for the lack of reproducibility of the model.

The use of lyophilized inoculum preparations, particularly when transferring CHIMs to disease-endemic regions, would help mitigate differences in reproducibility. The data from the *S. sonnei* 53G CHIM has shown that each new lyophilized inoculum stock needs to be clinically characterized for the desired AR in different settings; *S. sonnei* 53G dose was 1500 CFU when lyophilized or 400–500 CFU when frozen in North America, whereas a rather different frozen inoculum dose, 1600 CFU, was used in Thailand. Future CHIMs, regardless of geographical location, should implement lyophilized challenge strains as this standardization will be key to unraveling other factors important in either susceptibility or resistance to infection and allow better comparison between studies conducted in naïve versus exposed populations.

*Shigella*-CHIMs are now being translated to Kenya, where the regulatory framework already exists due to the ongoing malaria-CHIMs (Kapulu et al. 2018). Field trials of the leading *Shigella* vaccine candidates are ongoing in Kenya, making Kenya a logical place for the establishment of a *Shigella*-CHIM to allow direct comparison of experimental and field infection as well as generating evidence of cross-protection. Initially, the established *S. sonnei* 53G (lyophilized) model will be implemented (Wellcome and grants awarded 2005), but extending other *Shigella* CHIMs to this area is warranted. Several stakeholder consultations have been held to consider the ethical and regulatory frameworks for setup and conduct of CHIMs in the LMIC setting (Gordon et al. 2017; Elliott et al. 2018; Kunda-Ng’andu et al. 2021; Kapumba et al. 2020). While each region, country and setting are unique, common themes have emerged, highlighting the need to work closely with ethicists and regulators at the national and local levels. When considering a CHIM in an endemic country, the embedding of social science and empirical ethics substudies to highlight ethical dilemmas, perceptions and experiences for participation are required (Njue et al. 2018; Jao et al. 2020).

### 5.3 Expanding Shigella-CHIMs Beyond the Currently Established Serotypes

Typically, CHIMs use a single available challenge strain for each *Shigella* serotype, which should adequately represent the strain(s) circulating in the setting where the infection occurs. This however may pose some obvious complications as often many serotypes of a disease-causing strain exist in the field (e.g., influenza, pneumococcus, *Shigella*). Nevertheless, the use of a single-strain CHIM may still be meaningful in de-risking a multivalent vaccine candidate. In any case, caution should be exercised when extending results from one strain to other strains in order to avoid over-estimation of protection (Sack et al. 1994; Kerneis et al. 2009). Choice of the challenge strain may be influenced by epidemiology, need for homologous or heterologous protection studies and (presumed) mechanism of vaccine-conferred protection (antibody or cell-mediated; mucosal or systemic).

It is generally accepted that demonstration of safety, immunogenicity and efficacy will be required in the target population for a novel *Shigella* vaccine. The existing *S. sonnei* and *S. flexneri* 2a CHIMs can predict homotypic protection and may also be useful in the evaluation of multivalent *Shigella* vaccine candidates. However, the current epidemiology of other *S. flexneri* serotypes, targeted by multivalent vaccine candidates, may not support feasible field efficacy studies, in which case new *S. flexneri* serotype challenge strains may be useful for early evaluations as well as heterotypic protection resulting from immunologic cross reactivity. Thus, there is an urgent need to develop additional challenge strains of *Shigella* including but not limited to *S. flexneri* 3a and *S. flexneri* 6 which are currently described as globally important and relevant, especially in LMIC settings but are of lower prevalence compared with *S. sonnei* and *S. flexneri* 2a (Livio et al. 2014). To this end, a GMP lyophilized lot of *S. flexneri* 3a J17B is being manufactured by Walter Reed Army Institute of Research and, after identifying a dose that safely and reproducibly induces an AR of * 60–70%, it will be made available to the global community (Kaminski, personal communication).

Earlier efforts to develop a Shiga toxin mutant of *S. dysenteriae* for future vaccine studies should also be re-considered (Samandari et al. 2000) because this serotype is sporadically associated with increased morbidity and mortality during epidemic outbreaks. Similarly, the use of existing attenuated *Shigella* vaccine strains, in a broader sense, might enable CHIMs to be conducted in an outpatient setting due to their reduced fitness, enhanced safety and lower transmissibility. Collectively, these additional challenge strains may enable evaluation of vaccine efficacy against different *Shigella* species and serotypes for which current epidemiology limits the feasibility of classical Phase 3 efficacy studies. Additional *Shigella* CHIMs would also further aid the interrogation of heterologous protection and definition of cross-strain correlates of protective immunity.

The relationship between the safety profile of the CHIM and virulence of the challenge agent requires careful balance. The use of attenuated strains or live-attenuated vaccine pathogens in CHIMs can be useful in achieving this balance, especially when used in populations with increased disease susceptibility. This approach can reduce risks associated with severe disease complications and has been employed for viremia (e.g., dengue). Challenge agents may be genetically modified (naturally or via laboratory manipulation) to partially attenuate their activity as long as the changes do not dramatically impact the infectious process or the desired CHIM endpoint, thus restricting the adequate extrapolation of results into clinical settings. For example, the need to retain a viable pathogen is paramount if the intervention is believed to impact organism replication, dissemination within the host (cell to cell spread) and reduction or elimination of diseases. However, if the intervention is believed to act via transmission blocking or generation of sterile immunity, an attenuated challenge agent may be sufficient.

Likely, this narrow balance between strain attenuation and potential CHIM outcomes must be determined experimentally. Appropriate attenuated vaccine strains have been used as challenge agents to assess the efficacy of subunit vaccines (e.g., rotavirus) (Chilengi et al. 2020), and although a promising approach, there is no guarantee that protection against an attenuated strain will necessarily lead to protection against the wild-type strain. An additional complexity is that a naturally attenuated challenge strain may be considered similar to a genetically modified organism from a regulatory perspective and require higher levels of biological containment. An additional environmental risk assessment may be required to decide on contained use or level of physical isolation for use.

In the interest of model standardization, it has been suggested to generate well-characterized challenge strain banks. While bringing benefits of reproducibility and thus comparability between cohorts and study centers, it also requires that the strains have undergone extensive ex vivo manipulation and long-term storage (sometimes for decades) which may raise concerns around their representativeness of strains circulating in the field.

### 5.4 *Feasibility of Phase 3 Trials and Role of CHIMs in* Shigella *Vaccine Licensure and Full Value of Vaccine Assessments*

Like any in vivo model system, CHIMs represent a sophisticated opportunity to assess host–pathogen interactions. However, there are limitations including small sample sizes, selected from within a very specific population. This means results may lack external validity. The small sample size of CHIMs limits the statistical power to detect only the most overt differences. Additionally, while the lack of efficacy in a CHIM may discourage further evaluation of the candidate vaccine, demonstration of efficacy in a CHIM likely does not preclude the need for a field-based efficacy trial to confirm the CHIM results and allow licensure and policy recommendations.

There are only a limited number of viral or bacterial CHIMs that have been confirmed against real-life or field efficacy studies (Memoli et al. 2015; Darton et al. 2016). Typically, CHIMs use a single available challenge strain, which should adequately represent the strain(s) circulating in the setting where the infection occurs. This however poses some obvious challenges for *Shigella* as several serotypes cause disease in the field, whereas only two CHIMs have been established for *S*. *flexneri* 2a and *S. sonnei*. The reliability and validity of potential immunological CoP between CHIM serotypes are unknown between the two *Shigella* strains used in experimental infections (Giersing et al. 2019). Thus for interventions with proposed broad coverage against multiple serotypes, at least some efficacy will need to be (1) generated in field trials, in the target population, (2) extrapolated across strains based on field data, or (3) demonstrated in large randomized control Phase 3 trials. Well-developed and well-characterized CHIMs are a precious tool to assist in identifying the most promising candidates, defining immunological readouts relevant for clinical protection (Clarkson et al. 2021a) and providing insight for additional field studies to validate CHIM data and investment in candidate development (WHO 2020; MacLennan et al. 2019a).

There is no general acceptance or explicit guidance documents from regulators on the use of CHIM data in the licensure pathway for a vaccine (WHO 2016, 2017b; Ramanathan et al. 2019). Initially, CHIMs are unlikely to replace large Phase 3 trials, particularly for pediatric LMIC indications. If CHIM data are used to accelerate licensure, there is the implicit expectation that effectiveness studies will be completed post-licensure. CHIM data have supported US Food and Drug Administration (FDA) licensure of a cholera vaccine (Vaxchora ®) for the traveler’s market (Mosley et al. 2017). In this case, field trials were very difficult to perform due to the low incidence rate of seasonal disease in the target populations, and the inability to predict the timing and location of outbreaks. CHIMs demonstrated > 90% protective efficacy 10 days after vaccination and nearly 80% after 3 months. This, along with demonstrated safety in a larger cohort and confirmed immune responses, was sufficient for licensure in healthy adults at risk of contracting the disease during travel to highly endemic areas. Alternatively, *Salmonella Typhi* CHIM data were considered as a factor in the WHO policy recommendation of typhoid conjugate vaccines (TCV) (WHO 2017b; Burki 2018). In this instance, there was no good animal model available and the CHIM provided valuable information on the immune response and a serologic bridge to an effective licensed vaccine.

A key aspect of the full value of vaccines assessment is to de-risk and incentivize investment in vaccine development and testing. By reducing the reliance on large phase 3 efficacy studies, CHIMs can reduce both development timelines and cost. Positive efficacy results of a single *Shigella* serotype candidate vaccine in a homologous CHIM may support intervention selection, generate early efficacy signals and contribute to higher success rate of late-stage clinical development. Successful evaluation of a single *Shigella* serotype candidate vaccine in a homologous CHIM may also help to benchmark expected immune responses in a multivalent candidate vaccine.

### 5.5 Expanding CHIMs to Other Age Groups

In selected cases, CHIMs may also include vulnerable populations (e.g., immunocompromised, elderly, young children or pregnant women) to assess, in well-controlled situations, CoP and in some cases efficacy that may differ in these populations (e.g., elderly) compared to healthy young adults typically enrolled in CHIMs. Previous arguments have been made to support the extension of *Shigella*-CHIMs to children in endemic areas, as they are likely to encounter *Shigella* normally (Giersing et al. 2019). However, the potential risks of childhood experimental challenges currently outweigh any potential benefits, and as a result, *Shigella*-CHIM development in young children is not recommended. Early efficacy indication of treatment in elderly adults with reduced ability to mount appropriate immune responses might be warranted when balancing the risks associated with a CHIM and the risks associated with large, lengthy and costly field trials. Presently, National Regulatory Agencies in the US (FDA) and in Europe (European Medicine Agency, EMA) do not support the conduct of CHIMs in pediatric populations, pregnant women or immunocompromised subjects (FDA 2011; EMA 2018).

### 5.6 Expanding CHIMs for Use Beyond Vaccines

Besides their use in vaccine development, CHIMs can be very useful in the evaluation of other prevention or treatment modalities and can contribute to a better understanding of naturally acquired immunity when conducted in exposed populations. For the former, *Shigella*-CHIMs have demonstrated that rifaximin can be used prophylactically to prevent illness after challenge (Taylor et al. 2006; Tacket et al. 1992). As the *Shigella*-CHIMs are translated to exposed populations, they may be useful tools in unraveling and identifying key pathogen-specific antigen targets and host immune signatures that will inform second and third-generation *Shigella* vaccines. In addition, CHIMs can assist in the evaluation of other therapeutic products, including but not limited to prophylactics, antibiotics and phages-targeting *Shigella* species (Bernasconi et al. 2018; Shahin et al. 2020). In the era of innovative technological advancements, CHIMs will be used for the discovery of therapeutics such as pathogen-specific mAbs in addition to their subsequent testing for efficacy. The potential for new therapeutic solutions, such as mAbs, should have a high impact on combating the growing threat of AMR strains.

## 6 Outlook

The acquisition of natural immunity following repeated infections with *Shigella* suggests that a vaccine is feasible. However, the large number of *Shigella* serotypes complicates the development of a broadly protective vaccine. Thus, a vaccine must contain either multiple components, if based on the serogroup-specific OAg or several highly conserved protein antigens with varied virulence targets. Currently, several diverse technologies are being applied (alone or in combination) to *Shigella* vaccine development (Table 1), including novel glycoconjugation techniques (bioconjugation or synthetic conjugation), subunit protein approaches, GMMA technology (outer membrane exosomes) and live-attenuated strains. These approaches may well have differing abilities to potentiate immune responses in the pediatric age group as a result of their modes of delivery, antigen presentation and different mechanisms of action. *Shigella*-CHIMs will help to clarify the mechanism by which immunity is generated and provide insight into which platform may elicit the most appropriate immune responses.

The recent *Shigella*-CHIM efforts to better grow and deliver strains, assess the impact of pre-existing immunity on outcomes and develop more standardized clinical endpoints are initial steps needed to make *Shigella*-CHIMs a better tool, supporting product development and licensure. However, there is more to be done to expand the application of CHIMs, the strain serotypes that can be studied as well as the potential for antigen discovery and identification of CoP. Each of these contributes to maximizing the potential and value of multivalent *Shigella* vaccine candidates in development and possible combination vaccines that include multiple *Shigella* components. To achieve this, aspects of the CHIMs’ development and application need to be further explored to enable the platform to reach its full potential aiding both basic and applied research supporting the ongoing international *Shigella* prevention and control efforts.

### 6.1 Role of CHIMs in Vaccine Development

The high global *Shigella* burden and difficulty in effectively treating shigellosis has heightened interest in accelerating the development of new vaccines. The growing interest in accelerating innovative vaccine development has led to a number of international convening’s that have critically evaluated the fundamental processes by which vaccines are traditionally developed (Giersing et al. 2019). The major driver for this assessment is that historically there has been a low probability of success for candidate vaccines entering advanced clinical development. As a result, better tools are needed to de-risk projects by more rapidly identifying the better vaccine candidates. Current timelines for vaccine development are lengthy and often vaccines that reach Phase 3 field trials, if feasible, fail to provide the desired level of protective efficacy. The broader application of an experimental medicine platform that expands the use of experimental human models of infection and disease, used at the appropriate stages of development, could accelerate candidate vaccines and better ensure that only candidates with the highest probability of success advance to costly Phase 3 testing. There is now growing consensus that expanded use of CHIMs for enteric pathogens could be a potential paradigm shift and significantly shorten timelines of vaccines to licensure and WHO pre-qualification. For the most common bacterial cases of diarrhea, dysentery and enteric fever among young children and infants in LMICs and international travelers, CHIMs have for the most part been established and utilized in the US and to a lesser extent in Europe. The utility of CHIMs in accelerating vaccine development has previously been demonstrated through its central role in the recent FDA licensure of the Vaxchora oral cholera vaccine (Mosley et al. 2017) and WHO’s pre-qualification of Typbar-TCV, a TCV, for typhoid fever (Jin et al. 2017).

International conferences on CHIMs sponsored by IABS (Pollard et al. 2020), WHO and the Wellcome Trust in 2017 and more recently by the United Kingdom’s Academy for Medical Sciences in 2018 (AMS 2005) have all reaffirmed the pivotal importance of CHIMs in vaccine development, being particularly useful in early “proof of concept” efficacy studies (Gordon et al. 2017). These international workshops identified a number of issues that were pertinent to further maximize the impact of CHIMs on the development of new preventive and treatment interventions for shigellosis. Paramount among these was the recommendation to expand and build capacity to perform CHIMs in LIMCs with an important rationale being that research conducted within areas of unmet medical needs will result in outcomes more relevant to the at-risk population while boosting the quality of research conducted in these areas. These conferences also discussed the benefits and barriers to performing CHIMs in LMICs and identified the growing interest among LMIC investigators to explore the use of CHIMs in their respective countries to help in the development of new interventions that are more appropriate for use in their communities. These international meetings were a very important first step in expanding CHIMs application and the recent decision by the Wellcome Trust to support *Shigella*-CHIMs development in Kenya (Wellcome and grants awarded 2021) under the oversight of the US FDA and local regulatory authority is a clear indication that public health stakeholders appreciate the value of CHIM expansion to LMICs. To support the vaccine development paradigm shift, the models must be conducted using high research, regulatory and ethical standards.

### 6.2 Role of Challenge Strains

As the application of *Shigella* CHIMs expand, international stakeholders, such as WHO and the Wellcome Trust (Giersing et al. 2019; Wellcome Human infection studies for vaccine development 2021), have stressed the need for available *Shigella* challenge strains to support broad coverage vaccine development and other preventive interventions. To date, the 2457T strain of S. *flexneri* 2a and the 53G strain of *S. sonnei* have been the most commonly used challenge strains and only the *S. sonnei* 53G strain is available in a lyophilized form. Given that most *Shigella* vaccines under development are envisioned to be multivalent formations, providing coverage for at least three S. *flexneri* serotypes as well as S. *sonnei,* it will be useful that GMP challenge strains, preferably lyophilized, be prepared for at least *S. flexneri* 3a and S. *flexneri* 6 and their respective challenge doses and ARs be established in dose-ranging studies. *Shigella flexneri* 1b, 3a and 6 are also epidemiological important strains associated with diarrhea and dysentery among infants and children in LMICs but their overall incidence would make Phase 3 efficacy studies against these serotypes very difficult and costly to carry out. Thus, it is important to work in parallel to develop challenge strains for epidemiologically relevant *S. flexneri* serotypes to support the range of protection afforded by these multivalent vaccines. As new challenge strains are developed, it will also be important to evaluate strain delivery across serotypes to ensure optimal buffering and protection against gastric acid during the dosing process. Similarly, insights from recent human enteroid models and transcription studies on the role of factors impacting the expression of adhesins and other *Shigella* virulence factors (i.e., component of bile salts, sodium glycocholate hydrate) might serve to increase ARs and improve the reproducibility/reliability of the model, if they were incorporated into the growth media and/or delivery buffer of the *Shigella* challenge strains (Chanin et al. 2019; Morris et al. 2013).

### 6.3 Role in Understanding Markers of Inflammation and Innate Immunity

The significance of *Shigella* as an enteric pathogen with global public health impact has been enhanced in recent years by a growing awareness that infection and illness resulting from this bacterium contributes to the causal pathways for gut enteropathy, malnutrition, stunting and poor cognitive development (Guerrant et al. 2021; Kosek and Investigators 2017; Donowitz et al. 2018). This has increased interest in *Shigella* vaccines that may also reduce the level of intestinal colonization and both gut and systemic inflammation, given that both asymptomatic colonization and higher levels of inflammatory biomarkers, like C-reactive protein (CRP) and myeloperoxidase (MPO), have been associated with an increased risk of stunting and poor cognitive development among infants and young children in LMICs. With the increasing interest in *Shigella* vaccine impact on these secondary endpoints, CHIMs may enable an early signal of an effect. In addition to MPO and CRP, other markers of inflammation or gut health that should be considered for assessment in CHIMs include calprotectin, stool alpha anti-trypsin, plasma intestinal fatty acid-binding protein, plasma soluble CD14, soluble CD163, serum amyloid A protein and LPS-binding protein (Guerrant et al. 2021; Munoz et al. 1995; Kosek and Investigators 2017). All these markers could be easily measured at baseline, pre- and post-challenge over the course of the experimental infection period by more conventional methods and potentially by new multiplex technologies like multi-micronutrient and environmental enteric dysfunction assessment tool (MEEDAT) platform (Arndt et al. 2020). MEEDAT is a prototype assay to quantitate multiple markers of environmental enteric dysfunction, systemic inflammation, growth hormone resistance and micronutrient use in low-resource settings.

Although not generally considered as part of the assessment of post-challenge inflammation or activation of other innate immunity mediators, studies of *Shigella* pathogenesis have shown that in order for the bacteria to reach the large intestine, its site of infection, the bacteria must first pass through the stomach and small intestine. Highly effective acid resistance systems allow the bacteria to safely travel through the acidic environment within the stomach (Schroeder and Hilbi 2008; Gorden and Small 1993; Kararli 1995; Islam et al. 2001). Once the small intestine is reached, *Shigella* make use of host bile salts to induce the up-regulation of bacterial survival genes but also down-regulate the expression of host antimicrobial peptides, such as LL-37 and HBD-1, which are released at mucosal surfaces acting as barrier effectors to infection (Islam et al. 2001). Together, these virulence factors allow the safe passage of *Shigella* species through the stomach and small intestine, and they ultimately contribute to the low infectious dose observed with *Shigella* infections in nature, as well as documented in CHIMs, and to intestinal colonization and subsequent invasive enteric illness. In recent studies of a live-attenuated *Shigella* vaccine in adults and young children, it was observed that host defensins and antimicrobial peptides (e.g., LL-37 and HBD-1) did not increase post-vaccination in the youngest age group (Sarkar et al. 2021). However, they were elevated in adults, suggesting that this innate response might be accentuated in primed individuals and contribute to protection. Consequently, they should be further evaluated in CHIMs, and especially those conducted in exposed populations, where they could be easily measured along with other markers of inflammation and innate immune activation.

### 6.4 Role in Understanding Signatures of Immunity

The application of advanced Omics and systems biology technologies to better characterize host and bacterial interactions, in CHIMs of ETEC (Clarkson et al. 2021b; Ndungo et al. 2018; Crofts et al. 2018a, b; Chakraborty et al. 2019; Jin et al. 2021), *Campylobacter* (Crofts et al. 2018a, b; Chakraborty et al. 2019; Jin et al. 2021) and *Salmonella* Typhi (Clarkson et al. 2021b; Ndungo et al. 2018; Crofts et al. 2018a, b; Chakraborty et al. 2019; Jin et al. 2021), have given investigators greater insights into important antigens as well as innate and adaptive immune responses associated with protective immunity (Clarkson et al. 2021b; Ndungo et al. 2018; Crofts et al. 2018a, b; Chakraborty et al. 2019; Jin et al. 2021). These new analytical tools have not yet been applied to *Shigella*-CHIMs in a systematic way. With the planned expansion of *Shigella*-CHIMs, now would be an opportune time to integrate these technologies in the analysis plans for future trials as they would provide a unique opportunity to compare host and bacterial responses in individuals from developed countries versus those from Africa and potentially other LMIC sites. Early application of proteomic array analyses in *S. flexneri* 2a and *S. sonnei* CHIMs have already identified an immune signature or profile that may be predictive of protection, but this observation needs confirmation especially with other serotypes. Proteomic array studies may be particularly important in identifying threshold baseline levels of anti-OAg and/or anti-Ipa antibodies that are associated with protection in CHIMs. These observations may also help identify more relevant pre-existing antibody screening criteria that could better determine subject eligibility for enrolment in a CHIM and by so doing help to further ensure more reliable ARs for shigellosis.

The application of a systems serology approach to the analysis of samples from typhoid- and malaria-CHIMs has helped identify immune signatures associated with protection (Jin et al. 2021; Minassian et al. 2021). Recently, a system serology approach to analyze immune response profiles induced by experimental infection with *S. flexneri* 2a or *S. sonnei* strains has been described (Clarkson et al. 2021b), and this novel analysis has identified differences in immune response patterns induced by the two different species. These *Shigella* immune response profiles need to be further evaluated in future CHIMs because of their potential role in guiding vaccine development. *Shigella* heterotypic studies are currently planned that will examine cross-protection between *S. flexneri* 2a and *S. sonnei* induced by the initial infection with either serotype followed by challenge with the heterologous serotype (NCT04992520). The application of systems serology may be instrumental in defining the contribution of OAg epitopes and conserved protein antigens, like PSSP-1 (Kim et al. 2015) to cross-protective immunity.

### 6.5 Role in Evaluation of Combination Vaccines

Finally, WHO’s recently published preferred product characteristics for a *Shigella* vaccine notes that the full public health value proposition can be improved by inducing protection with fewer doses (WHO 2020) and combining delivery with other enteric vaccines, such as TCV. The various CHIMs are well suited to aid this development effort and may be critical to ensuring a greater impact for *Shigella* vaccines. Single-dose or novel prime-boost options (Keren et al. 1988; Hartman et al. 1994), as well as the value of adjuvants in improving vaccine-induced protection, can also be evaluated in CHIMs. With CHIMs now well-established for *Shigella* (Talaat et al. 2019; MacLennan et al. 2019b), ETEC (Crofts et al. 2018a), *Salmonella* Typhi and Paratyphi A (Dobinson et al. 2017; Waddington et al. 2014; Gibani et al. 2020), *Campylobacter* (Crofts et al. 2018b) and cholera (Shirley 2011), novel combination vaccines can be evaluated for efficacy early in clinical development so that the optimal pathogen and antigenic combinations can be advanced to Phase 3 trials and licensure.

### 6.6 Recap

Our appreciation of the high burden of *Shigella*-associated disease and the growing AMR threat among infants and children in LMIC has increased. Similarly, the role of *Shigella* infection as a potential trigger for both acute and longer-term functional bowel disorder in travelers has gained more widespread acceptance. There has also been growing consensus that the development of more effective treatments and preventative interventions for shigellosis needs to be accelerated. As a result, the number of promising *Shigella* vaccine candidates in the current pipeline is unprecedented. It is generally agreed among international public health stake-holders and donors that the *Shigella*-CHIMs will play a central role in fast-tracking the development of better prevention and control measures.

## Figures and Tables

**Table 1:** *Shigella* vaccines currently in clinical development^*^

Target	Serotype	Technology	Translational	Phase 1	Phase 2a	CHIM
Single Serotype	*S*. *flexneri* 2a	Bioconjugate		Flexyn2a ^a^ (LMTB) NCT02388009		Flexyn2a ^a^ (LMTB) NCT02646371
		Synthetic Conjugate		Sf2a-TT15 (IP) NCT02797236	Sf2a-TT15 (IP) *NCT04602975*	Sf2a-TT15 (IP) *NCT04078022*
		Invasin Complex		Invaplex_AR_ (WRAIR) NCT02445963		
				Invaplex_AR_-Detox (WRAIR) NCTO3869333		
	S. *sonnei*	GMMA		1790GAHB ^a^ (GSK) NCT02017899 NCT02034500 NCT03089879	1790GAHB ^a^ (GSK) NCT02676895	1790GAHB ^a^ (GSK) NCT03527173
		Live-Attenuated Oral		WRSs2 (WRAIR/NIAID) NCT01336699		WRSs2 (WRAIR/NIAID) *NCT04242264*
Multiple Serotypes	*S. sonnei* and *S*. *flexneri* 2a, 3a, 6	Bioconjugate		Shigella4V (LMTB) **NCT04056117**	Shigella4V (LMTB) **NCT04056117**	
	*S*. *sonnei* and *S*. *flexneri* 1b, 2a, 3a	GMMA	altSonflex1-2−3 (GSK) *NCT05073003*			
Multi-Pathogen	*S*. *flexneri* 2a, ETEC	Live-Attenuated Oral		CVD1208S-122 (UMD)		
				***NCT04634513***		
				Live-Attenuated Oral		ShigETEC (Eveliqure) Harutyunyan et al. (2020)		

a Single serotype candidate vaccine development terminated in favor of multi-serotype candidate vaccine

* **Trial status:** *Not yet recruiting/recruiting*; **Active/Ongoing;** Completed

**Product Developers** Eveliqure—Eveliqure Biotechnologies GmbH (Vienna, AT); GSK—GSK Vaccines Institute for Global Health (Siena, IT); IP—Institut Pasteur (Paris, FR); LMTB—LimmaTech Biotechnology (Zurich, CH); NIAID—National Institutes of Allergy and Infectious Diseases (Bethesda, MD, USA); UMD—University of Maryland (Baltimore, MD, USA); WRAIR—Walter Reed Army Institute of Research (Silver Spring, MD, USA)

**Table 2:** Utilization of *Shigella flexneri* 2a and **S.** *sonnei* controlled human infection models

Evaluation	Challenge agent	Product under evaluation	Naïve attack rate (n/N)	Treated attack rate (n/ N)	Efficacy (%) ^a^	References
Homologous re-challenge	*S. flexneri* 2a	Strain 2457T	22/39	3/15	64	DuPont et al. (1972b)
	*S. flexneri* 2a	Strain 2457T	11/12	3/11	71	Kotloff et al. (1995a)
	S. *sonnei*	Strain 53G (frozen)	8/12	0/6	100	Herrington et al. (1990)
Antibiotic	*S. flexneri* 2a	Rifaximin	6/15	0/15	100	Taylor et al. (2006)
	*S. flexneri* 2a	Rifaximin	13/15	n/a	n/a	Taylor et al. (2008)
Passive Ig	*S. flexneri* 2a	Bovine IgG	5/11	0/10	100	Tacket et al. (1992)
Homologous vaccine candidate	*S. flexneri* 2a **b**	Heat-killed whole cell	19/30	18/25	-14	Shaughnessy and Olsson (1946)
	*S. flexneri* 2a **b**	Irradiated whole cell	19/30	23/28	-30	Shaughnessy and Olsson (1946)
	*S. flexneri* 2a	EcSF2a-1(E. *coli K-12—S. flexneri* 2a OAg hybrid live-oral)	6/24, 52/88	1/15, 30/68	73, 25	DuPont et al. (1972b)
	*S. flexneri* 2a	Streptomycin-dependent whole cell	6/24, 52/88	3/31, 16/53	61, 49	DuPont et al. (1972b)
	*S. flexneri* 2a	EcSF2a-2 (*E. coli* K-12 aroD mutant—*S. flexneri* 2a OAg hybrid live-oral)	12/14	10/16	27	Kotloff et al. (1995b)
	*S. flexneri* 2a	SC602 (live attenuated S. *flexneri* 2a *icsA* mutant)	6/7	0/7	100	Coster et al. (1999)
	*S. flexneri* 2a	Proteosome-Sflex2a LPS	13/13	9/14	36	Durbin et al. (2001)
	*S. flexneri* 2a	Invaplex50 _NAT_ (Macromolecular complex containing IpaB, IpaC and *S. flexneri* 2a LPS)	8/12	7/10	-5	NCT 00485134
	*S. flexneri* 2a	Flexyn2a, bioconjugate (*S. flexneri 2a* OAg conjugated to *P. aeruginosa* exotoxin A)	18/29	13/30	30	Talaat et al. (2021)
	S. *sonnei* (Frozen)	WRSs1 (Live attenuated S. *sonnei virG* mutant)	1/6 ^c^	0/13	n/a ^c^	Pitisuttithum et al. (2016)
	*S. sonnei* (Lyophilized)	1790GAHB (S. *sonnei* GMMA-technology)	15/32	12/28	− 9.4	Frenck et al. (2021)

a Efficacy outcomes presented as the per protocol definition for shigellosis

b *S*. *flexneri* 2a strain was FW rather than the more commonly used 2457T

c Study performed in Thai adults yielded lower than anticipated naïve AR, and the authors reported “no efficacy against dysentery and diarrhea”

**Table 3:** Proportion of subjects developing symptoms or signs of disease in **S.** *flexneri* 2a (Porter et al. 2018) and **S.** *sonnei* CHIMs (Frenck et al. 2020)

Symptom/sign	**S.** *flexneri* 2a, 2457T	**S.** *sonnei,* 53G ^a^
Diarrhea	75.9	70.0
Fever	50.0	23.9
Nausea	46.3	30.4
Abdominal pain/cramps	81.5	69.6
Malaise	57.4	43.4
Headache	66.7	69.6
Myalgia	42.6	47.8
Arthralgia	20.4	26.1
Anorexia	64.8	41.3
Vomiting	24.1	19.6
Dysentery	27.8	43.4

a Excludes subjects receiving 500 CFU dose

**Table 4:** Conduct of *Shigella*-CHIMs in different population settings

CHIM setting	Pros	Cons
Traditional - in *Shigella* naïve subjects	Well controlled clean system, potentially increasing reproducibility Immunologically unprimed Has potential to be predictive of target populations	Minimal co-morbidities which would be present in endemic setting Differences in genetic background compared to target population
Next-generation - in *Shigella* exposed subjects	Closer representation of target population environmental variables Accounts for population genetics Accounts for the impact of prior exposure/immunologic priming/ baseline immunity May help to better define protection/efficacy and true CoP Cost-benefit Acceleration of interventions Capacity building	Availability of representative circulating strains as inoculum Sensitivity of the model to overcome pre-existing and variable immunity, potentially decreasing reproducibility Low AR, impacting ability to draw meaningful conclusions Differentiating vaccine efficacy from background immunity Infrastructure, clinical/ containment

## Text Box 1 Key features in the conduct of *Shigella-CHIMs*

The standardized methods for performing *Shigella* CHIMs are comprehensively presented in Talaat et al. (Talaat et al. 2019). *Shigella* CHIMs are exclusively conducted as inpatient studies due to the potential severity of illness and the need for careful monitoring, the clinical endpoints to be measured and transmissibility of *Shigella* (infection control).

### Bacterial challenge strains

*Shigella*-CHIMs have been established based upon historical and contemporary studies. Both *S. flexneri* 2a 2457T and *S. sonnei* 53G were isolated from patients with clinical disease in the 1950s (DuPont et al. 1969, 1989) and manufactured to GMP standards as frozen or lyophilized challenge agents, respectively, for administration to healthy volunteers. Both GMP-prepared cell banks are highly antibiotic sensitive and remain susceptible to ciprofloxacin and trimethoprim-sulfamethoxazole, which are commonly used to treat shigellosis.

### Challenge inoculum

Frozen or lyophilized challenge strains are used to prepare bacterial suspensions diluted to the defined target dose and delivered within 4 h. Results of proof-of-principle and dose-finding studies (Table 2) have led to the use of standard inoculum of 1500 colony forming units (CFU) for both *S. flexneri* 2a 2457T and *S. sonnei* 53G (lyophilized). For both CHIMs, this inoculum dose was selected for safety and to achieve the desired shigellosis attack rate (AR) of 60–70% in healthy volunteers aged 18–60 with no known prior history of shigellosis.

### Challenge administration

To improve the AR prior to the challenge, volunteers fast for at least 90 min and drink 100–120 mL of bicarbonate buffer to neutralize gastric acid (Kotloff et al. 1995a). Subsequently, they drink a 30 mL solution containing the *Shigella* challenge inoculum.

### Antibiotic therapy

Volunteers are drug treated once they meet the primary endpoint of shigellosis, have a fever ≥ 39 °C or at day 5 following administration of the challenge. All volunteers receive a 3–5 days treatment course and must have two consecutive *Shigella-*negative stool cultures prior to discharge.

## Text Box 2 Clinical endpoints used in *Shigella*-CHIMs

To enable comparison among sites conducting *Shigella*-CHIMs there have been recent efforts to harmonize primary and secondary clinical endpoints (Talaat et al. 2019; MacLennan et al. 2019b).

### Primary endpoints

- Severe diarrhea defined as ≥ 6 loose stools (grade 3 to 5) or > 800 g loose stools in a 24 h period
- Moderate diarrhea defined as 4–5 loose stools or 400–800 g loose stools in a 24 h period with accompanying signs or symptoms
- Dysentery defined as ≥ 2 loose hemoccult positive stools in a 24 h period with accompanying signs or symptoms
- Accompanying signs or symptoms include: oral temperature ≥ 38 °C or ≥ 1 moderate constitutional/enteric symptom (nausea, abdominal pain/cramps, myalgia/arthralgia, malaise) or ≥ 2 episodes of vomiting in a 24 h period.

### Secondary endpoints

- Percentage of volunteers with diarrhea, dysentery or fever
- Percentage of volunteers reporting moderate to severe nausea, vomiting, anorexia, gas or abdominal pain/cramps
- Percentage of volunteers requiring early antibiotic therapy or intravenous fluids
- Diarrhea characteristics including mean/median time to onset, mean/ median duration and maximum output total or in any 24 h period.
- *Shigella* disease severity score (see Sect. 2.6).
